# International multicenter validation of AI-driven ultrasound detection of ovarian cancer

**DOI:** 10.1038/s41591-024-03329-4

**Published:** 2025-01-02

**Authors:** Filip Christiansen, Emir Konuk, Adithya Raju Ganeshan, Robert Welch, Joana Palés Huix, Artur Czekierdowski, Francesco Paolo Giuseppe Leone, Lucia Anna Haak, Robert Fruscio, Adrius Gaurilcikas, Dorella Franchi, Daniela Fischerova, Elisa Mor, Luca Savelli, Maria Àngela Pascual, Marek Jerzy Kudla, Stefano Guerriero, Francesca Buonomo, Karina Liuba, Nina Montik, Juan Luis Alcázar, Ekaterini Domali, Nelinda Catherine P. Pangilinan, Chiara Carella, Maria Munaretto, Petra Saskova, Debora Verri, Chiara Visenzi, Pawel Herman, Kevin Smith, Elisabeth Epstein

**Affiliations:** 1 Department of Clinical Science and Education, Södersjukhuset, Karolinska Institutet, Stockholm, Sweden; 2 Department of Obstetrics and Gynecology, Södersjukhuset, Stockholm, Sweden; 3 School of Electrical Engineering and Computer Science, KTH Royal Institute of Technology, Stockholm, Sweden; 4 Science for Life Laboratory, Stockholm, Sweden; 5 Department of Gynecological Oncology and Gynecology, Medical University of Lublin, Lublin, Poland; 6 Unit of Obstetrics & Gynecology, Department of Biomedical and Clinical Sciences, Luigi Sacco University Hospital, University of Milan, Milan, Italy; 7 Institute for the Care of Mother and Child, Prague, Czech Republic; 8 Third Faculty of Medicine, Charles University, Prague, Czech Republic; 9 Department of Medicine and Surgery, University of Milan-Bicocca, Milan, Italy; 10 UO Gynecology, Fondazione IRCCS San Gerardo dei Tintori, Monza, Italy; 11 Department of Obstetrics and Gynaecology, Lithuanian University of Health Sciences, Kaunas, Lithuania; 12 Unit of Preventive Gynecology, European Institute of Oncology IRCCS, Milan, Italy; 13 Gynecologic Oncology Centre, Department of Gynecology, Obstetrics and Neonatology, First Faculty of Medicine, Charles University and General University Hospital in Prague, Prague, Czech Republic; 14 Fondazione Poliambulanza Istituto Ospedaliero, Brescia, Italy; 15 Obstetrics and Gynecology Unit, Forlì and Faenza Hospitals, AUSL Romagna, Forlì, Italy; 16 Department of Obstetrics, Gynecology, and Reproduction, Dexeus University Hospital, Barcelona, Spain; 17 Department of Perinatology and Oncological Gynecology, Faculty of Medical Sciences, Medical University of Silesia, Katowice, Poland; 18 Centro Integrato di Procreazione Medicalmente Assistita e Diagnostica Ostetrico-Ginecologica, Azienda Ospedaliero Universitaria-Policlinico Duilio Casula, Monserrato, University of Cagliari, Cagliari, Italy; 19 Institute for Maternal and Child Health, IRCCS ‘Burlo Garofolo’, Trieste, Italy; 20 Department of Obstetrics and Gynecology, Skåne University Hospital, Lund, Sweden; 21 Section of Obstetrics and Gynecology, Department of Clinical Sciences, Università Politecnica delle Marche, Azienda Ospedaliero-Universitaria delle Marche, Ancona, Italy; 22 Department of Obstetrics and Gynecology, Clínica Universidad de Navarra, Pamplona, Spain; 23 First Department of Obstetrics and Gynecology, Alexandra Hospital, Medical School, National and Kapodistrian University of Athens, Athens, Greece; 24 Department of Obstetrics and Gynecology, Rizal Medical Center, Manila, Philippines; 25 Gynecologic and Obstetric Unit, Women’s and Children’s Department, Forlì Hospital, Forlì, Italy; 26 Gynecology and Breast Care Center, Mater Olbia Hospital, Olbia, Italy; 27 Digital Futures, KTH Royal Institute of Technology, Stockholm, Sweden

**Keywords:** Cancer imaging, Ovarian cancer, Ultrasonography, Machine learning, Diagnosis

## Abstract

Ovarian lesions are common and often incidentally detected. A critical shortage of expert ultrasound examiners has raised concerns of unnecessary interventions and delayed cancer diagnoses. Deep learning has shown promising results in the detection of ovarian cancer in ultrasound images; however, external validation is lacking. In this international multicenter retrospective study, we developed and validated transformer-based neural network models using a comprehensive dataset of 17,119 ultrasound images from 3,652 patients across 20 centers in eight countries. Using a leave-one-center-out cross-validation scheme, for each center in turn, we trained a model using data from the remaining centers. The models demonstrated robust performance across centers, ultrasound systems, histological diagnoses and patient age groups, significantly outperforming both expert and non-expert examiners on all evaluated metrics, namely F1 score, sensitivity, specificity, accuracy, Cohen’s kappa, Matthew’s correlation coefficient, diagnostic odds ratio and Youden’s J statistic. Furthermore, in a retrospective triage simulation, artificial intelligence (AI)-driven diagnostic support reduced referrals to experts by 63% while significantly surpassing the diagnostic performance of the current practice. These results show that transformer-based models exhibit strong generalization and above human expert-level diagnostic accuracy, with the potential to alleviate the shortage of expert ultrasound examiners and improve patient outcomes.

## Abstract (web summary)

In a comprehensive dataset from 3,652 patients across 20 centers in eight countries, an ultrasound-based AI model shows robust performance across centers, ultrasound systems, 58 histological diagnoses and patient age groups and reduced referral to experts by 63% in a retrospective triage simulation.

## Main

Ovarian tumors are common and often incidentally detected. Their management depends on the estimated risk of malignancy and patient symptoms. Patients with a presumably benign lesion are generally managed conservatively with ultrasound follow-up or, if symptomatic, with minimally invasive surgery at a regional hospital to preserve fertility, avoid unnecessary costs and reduce morbidity^1,2^. Patients with suspected ovarian cancer benefit from referral to a gynecologic oncologist, as surgical expertise improves their chances of survival^3,4^.

Transvaginal ultrasound examination is the primary technique used to differentiate between benign and malignant ovarian lesions due to its wide availability and high diagnostic accuracy when performed by an experienced examiner^5,6^. However, the diagnostic accuracy and interobserver agreement tend to be considerably lower among less experienced examiners, which can result in delayed and incorrect cancer diagnoses, as well as unnecessary treatment^7,8^. Biopsy is contraindicated as it may cause a malignant tumor to spread, worsening the prognosis^3^. Unfortunately, even in high-income countries, there is a substantial lack of expert ultrasound examiners, leading to delayed and missed diagnoses, thus putting a substantial burden on the healthcare system.

Artificial intelligence (AI)-driven diagnostic support is a potential solution, and it has previously been shown that neural networks with convolutional neural network (CNN) architectures yield promising results in the classification of ovarian lesions^9,10^. However, a common pitfall in medical AI research, especially when using retrospective data, is the practice of training and evaluating models on data from the same distribution, that is, data that is homogenous in content and characteristics^11^. Practitioners often assume that unseen data will have the same distribution as the samples on which their models were trained^12^. This is rarely the case in clinical practice, as clinical environments are highly variable, and factors such as patient populations, imaging devices and acquisition protocols can differ substantially between centers^11^. Furthermore, the collection of datasets that are large and diverse enough to capture the full range of variability in clinical data and be universally representative is limited by both legal and economic constraints. This limitation can contribute to what is known as ‘domain shift’, where the data a model encounters when deployed in a clinical setting differ from the data it was trained on^13–15^. Failure to adequately address this can lead to poor performance, as the model may be unable to adapt to variations in new, unseen data not captured in the training data^11^. A recent meta-analysis found that most studies comparing healthcare professionals and AI models fail to properly validate performance using external data^16^, leading to a systematic overestimation of diagnostic accuracy in the scientific literature. Therefore, as researchers have increasingly pointed out, it is crucial to thoroughly evaluate a model’s ability to generalize to new populations and settings^17,18^. A large-scale multicenter study validating generalizability could provide essential evidence that boosts trust and confidence in AI-driven diagnostic support systems for clinical use.

In this international multicenter retrospective study, the Ovarian tumor Machine Learning Collaboration - Retrospective Study (OMLC-RS), we assessed the ability of neural networks to distinguish between benign and malignant ovarian tumors in ultrasound images, using a comprehensive dataset of 17,119 ultrasound images from 3,652 patients across 20 centers in eight countries, acquired using 21 different ultrasound systems from nine manufacturers. We used a state-of-the-art transformer-based model architecture^19,20^, which has been shown to be a competitive alternative to CNNs for medical imaging tasks^21,22^. Using a leave-one-center-out cross-validation scheme, for each center in turn, we trained a model using the data from the remaining centers. With each model trained in a similar fashion, we evaluated their ability to generalize across different patient populations, centers and ultrasound systems and compared their diagnostic performance with that of 66 human examiners with varying levels of expertise. We further simulated and assessed the integration of an AI-assisted triage strategy into routine clinical practice, with the aim of improving diagnostic accuracy and reducing human resource demands (that is, the number of examinations needed to make a management decision).

## Results

### AI models significantly outperform human expert examiners

**Extended Data Fig. 1:**
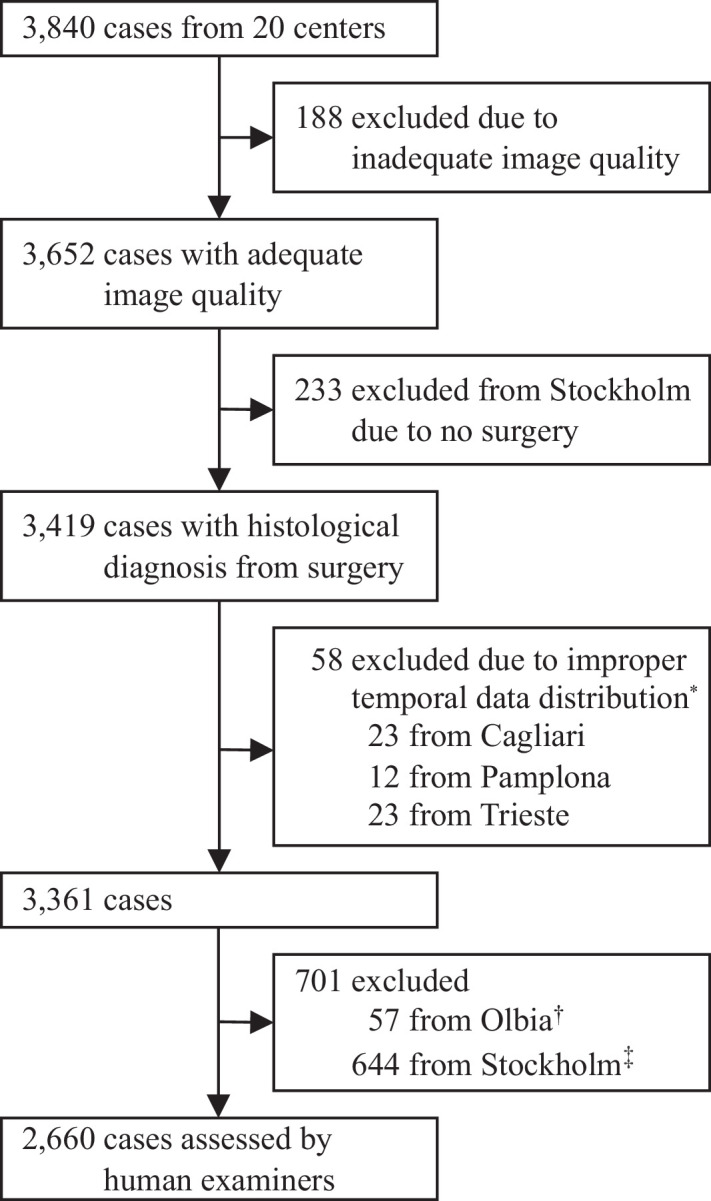
Study flow diagram. ^*^These cases were excluded from the main analysis as they had not been included in compliance with our criterion on the temporal distribution of examination dates. ^†^ The Olbia center was excluded from the human review due to its limited sample size (*n* = 57) and its small number of malignant cases (*n* = 8). ^‡^ These cases were excluded in order to have a test set of comparable size (*n* = 300) to those of the other centers and to utilize our reviewer resources efficiently.

The OMLC-RS dataset was used to train a series of 19 transformer-based neural network models (one model per center, except for one center that was excluded due to its limited sample size; Methods)^20^. We applied a leave-one-center-out cross-validation scheme, where iteratively each center in turn was isolated as the test set and the model was given the cases from the remaining centers for training. To establish a meaningful reference for comparison, we collected a total of 51,179 assessments from 33 expert and 33 non-expert examiners. Out of the 3,652 cases in the OMLC-RS dataset, each of 2,660 cases was assessed by at least seven expert and six non-expert examiners. The remaining 992 cases were used as supplementary training data (Methods and Extended Data Fig. 1).

**Fig. 1:**
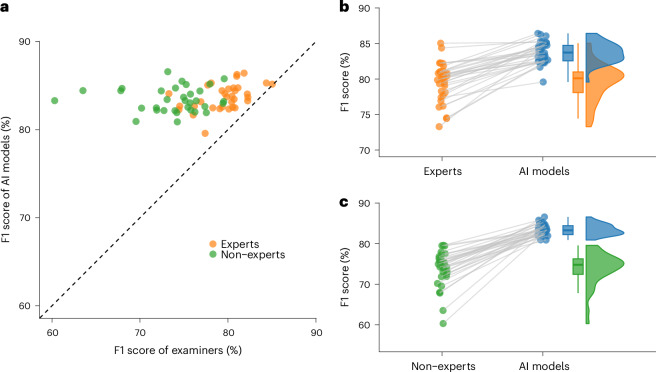
Paired F1 scores between human examiners and AI models. **a**, Paired F1 scores between individual examiners (*n* = 66) and the AI models on matched case sets, that is, each examiner is compared against the AI models on the set of cases he or she assessed. A dot above the dashed line corresponds to an individual examiner that was outperformed by the AI models on the same set of cases. **b**,**c**, Paired F1 scores between (**b**) expert examiners (*n* = 33; orange) and AI models (blue), and (**c**) non-expert examiners (*n* = 33; green) and AI models (blue), with gray lines indicating matched case sets. The box plots show the median and the 25th and 75th percentiles, and the whiskers span the range of non-outlier values. The density plots show the distributions of the overall F1 scores (made with kernel smoothing).

**Fig. 2:**
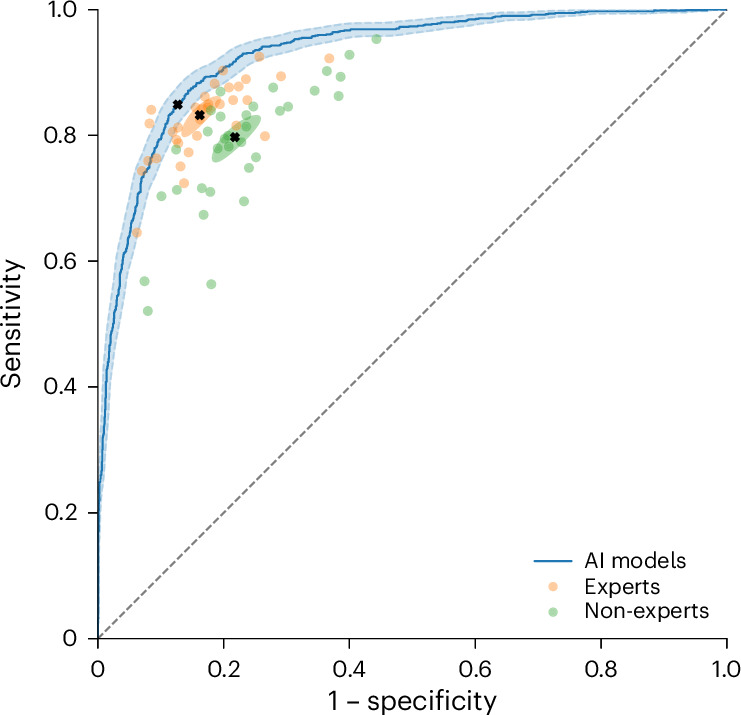
AI model ROC curve and human examiner performance. The model performance is given as an ROC curve in blue, with shaded 95% confidence bands constructed from the 2.5th and 97.5th percentiles of sensitivity values, at each level of specificity, from bootstrapped ROC curves. Each dot represents a human examiner, with non-experts in green and experts in orange. The performance of the AI models at the default cutoff point of 0.5, and the mean performance for expert and non-expert examiners, are each marked by a black cross. The mean performance for expert and non-expert examiners are each surrounded by a shaded 95% confidence region, estimated by a bivariate random-effects model^39^. Note that the models were evaluated on all 2,660 reviewed cases, but each individual examiner assessed only a subset of these cases. Hence, although multiple individual expert examiners seem to outperform, or perform on par with the models, by being positioned above or to the left of the ROC curve of the models, no examiner outperformed the models on the same case set, which can be seen in Fig. 1 and Supplementary Table 2.

We evaluated the models by comparing their diagnostic performance against expert and non-expert examiners on ultrasound images from these 2,660 patients with an ovarian lesion (1,575 benign and 1,085 malignant, according to histological assessment from surgery within 120 days of their ultrasound assessment; Table 1) at 19 centers in eight countries. The diagnostic performance, expressed as accuracy, sensitivity, specificity, F1 score, Cohen’s kappa coefficient, Matthew’s correlation coefficient (MCC), diagnostic odds ratio (DOR) and Youden’s J statistic, is shown in Table 2. We used the F1 score as the primary metric when comparing the models to human examiners as it provides a balance between precision and recall. The models outperformed both expert and non-expert examiners (*P* < 0.0001; Supplementary Table 1), which is consistent for all evaluated metrics. The paired F1 scores between each human examiner and the AI models show that the models achieved higher F1 scores than each of the 66 human examiners (Fig. 1), which is true also for accuracy, Cohen’s kappa, MCC and Youden’s J statistic. The diagnostic performance of the individual human examiners, with the corresponding scores for the AI models on matching case sets, can be found in Supplementary Table 2. The models achieved an F1 score of 83.50% (95% CI, 81.76–85.14) on cases from unseen centers, outperforming both expert and non-expert examiners, with F1 scores of 79.50% (95% CI, 77.57–81.19; Δ = 4.00 (95% CI, 2.34–5.83, *P* < 0.0001)) and 74.10% (95% CI, 72.05–76.09; Δ = 9.40 (95% CI, 7.46–11.35, *P* < 0.0001)), respectively. The difference in diagnostic error rates between the AI models and expert examiners is similar to that between expert and non-expert examiners. The false negative rate (FNR; 1 – sensitivity) and false positive rate (FPR; 1 – specificity) for the AI models are respectively 14.14% (15.12% versus 17.60%) and 26.74% (12.70% versus 17.33%) lower than those of the expert examiners. For comparison, the relative differences in FNR and FPR between expert and non-expert examiners are 17.32% (17.60% versus 21.29%) and 23.74% (17.33% versus 22.73%), respectively. For the AI models and the non-expert examiners, the relative differences in FNR and FPR are much larger at 29.00% (15.12% versus 21.29%) and 44.13% (12.70% versus 22.73%), respectively. The receiver operating characteristic (ROC) curve (Fig. 2) illustrates that the AI models outperformed both mean expert and non-expert performance over a range of potential cutoff points.

**Table 1:** Categories of histological diagnoses

	All data (*n* = 3,652)	Test data (*n* = 2,660)
**Benign**	2,224 (60.9%)	1,575 (59.2%)
Endometrioma	336 (9.2%)	276 (10.4%)
Dermoid	431 (11.8%)	340 (12.8%)
Other common benign	298 (8.2%)	222 (8.3%)
Solid benign	153 (4.2%)	118 (4.4%)
Cystadeno(fibro)ma	707 (19.4%)	569 (21.4%)
Rare benign^a^	66 (1.8%)	50 (1.9%)
Ultrasound follow-up^a^	233 (6.4%)	0 (0.0%)
**Malignant**	1,428 (39.1%)	1,085 (40.8%)
Borderline (serous)	207 (5.7%)	160 (6.0%)
Borderline (mucinous intestinal)	100 (2.7%)	79 (3.0%)
Ovarian cancer (epithelial)	804 (22.0%)	611 (23.0%)
Ovarian cancer (nonepithelial)	116 (3.2%)	89 (3.3%)
Metastasis	201 (5.5%)	146 (5.5%)

Counts are accompanied by their total percentage rate. The test data are the subset of the dataset included in the human review.

^a^For training, rare benign (*n* = 66) and ultrasound follow-up (*n* = 233) cases were, when possible, assigned to one of the five benign histological classes, based on the sonographic characteristics (as assessed by one expert examiner (E.E.)).

**Table 2:** Performance of AI models, human examiners and triage strategies

	Human resources	F1 score	Sensitivity	Specificity	Accuracy	Kappa	MCC	DOR	J
Single non-expert	1	74.10% (72.05–76.09)	78.71% (75.93–80.89)	77.27% (75.11–79.21)	77.67% (76.09–79.25)	0.546 (0.513–0.578)	0.548 (0.516–0.580)	12.26 (10.20–14.91)	55.51% (52.29–58.80)
Single expert	1	79.50% (77.57–81.19)	82.40% (80.08–84.51)	82.67% (80.89–84.61)	82.63% (81.17–84.02)	0.645 (0.614–0.673)	0.646 (0.615–0.674)	22.63 (18.41–27.54)	65.24% (62.17–67.96)
Current practice	1.52 (1.50–1.54)	77.16% (75.16–79.18)	73.82% (71.20–76.46)	87.94% (86.36–89.53)	82.18% (80.71–83.65)	0.626 (0.595–0.657)	0.628 (0.597–0.659)	20.53 (16.89–25.46)	61.73% (58.67–64.88)
AI models alone	1	83.50% (81.76–85.14)	84.88% (82.73–86.96)	87.30% (85.66–88.94)	86.32% (85.00–87.59)	0.718 (0.691–0.745)	0.718 (0.691–0.745)	38.61 (31.16–48.74)	72.19% (69.46–74.86)
AI-assisted non-expert	1.19 (1.18–1.21)	82.70% (80.99–84.37)	85.81% (83.65–87.78)	85.08% (83.33–86.84)	85.38% (84.02–86.69)	0.700 (0.673–0.728)	0.702 (0.674–0.729)	34.39 (27.83–43.32)	70.84% (68.11–73.57)
AI-assisted expert	1.15 (1.13–1.16)	83.56% (81.94–85.20)	85.99% (83.97–88.06)	86.29% (84.65–88.03)	86.20% (84.92–87.52)	0.717 (0.691–0.744)	0.718 (0.692–0.745)	38.80 (31.61–49.38)	72.33% (69.76–75.06)

Data in parentheses are 95% CIs. Human resources are the number of examinations needed to make a management decision. Kappa = Cohen’s kappa coefficient. J = Youden’s J statistic. Current practice = non-expert examiner with referral to expert in uncertain or presumably malignant cases; AI-assisted (non-)expert = AI model and (non-)expert consensus, with referral to (second) expert in cases of disagreement. See Fig. 4 for details.

### Sensitivity and specificity

To directly compare the sensitivity and specificity of the AI models with that of the expert and non-expert examiners, we also present the performance of the models at matching cutoff points (Extended Data Table 1). Our findings reveal that the AI models exhibit superior sensitivity (89.31% versus 82.40%; Δ = 6.91 (95% CI, 4.67–9.26, *P* < 0.0001)) when specificity is held constant at the expert level (82.67%). This corresponds to a 39.27% reduction in FNR with respect to expert examiners. They also excel in specificity (88.83% versus 82.67%; Δ = 6.16 (95% CI, 4.29–7.80, *P* < 0.0001)) when sensitivity is set at the expert level (82.40%) (Extended Data Table 1), corresponding to a 35.53% reduction in FPR. When compared to non-expert examiners, the disparities are even more substantial, with differences of 13.92 (95% CI, 11.74–16.70) and 13.27 (95% CI, 11.53–15.47) percentage points in sensitivity and specificity, respectively (Extended Data Table 1), corresponding to a reduction in FNR and FPR of 65.37% and 58.38% with respect to non-expert examiners.

### Subgroup analysis

**Fig. 3:**
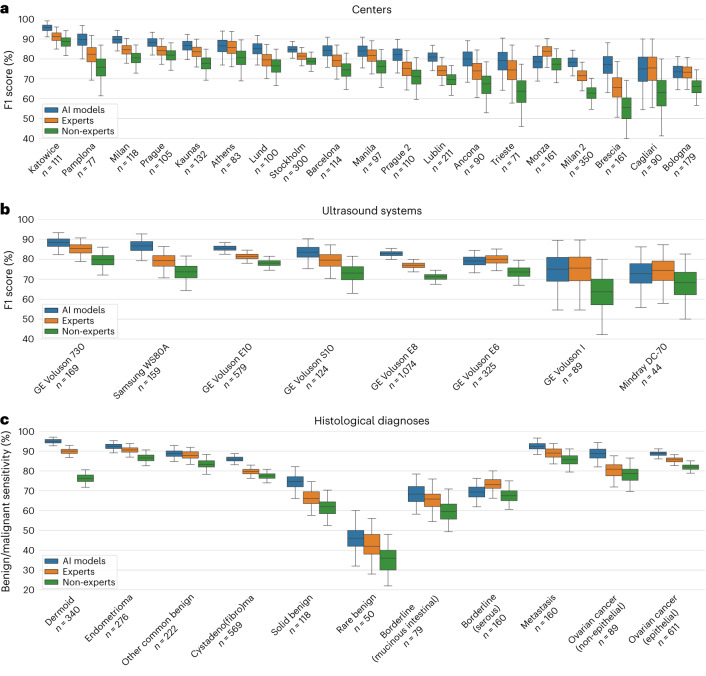
Subgroup analysis. **a**–**c**, Comparison of the AI models and expert and non-expert examiners, for different (**a**) medical centers, (**b**) ultrasound systems (limited to the eight most common systems), and (**c**) histological diagnoses. The box plots show the median and the 25^th^ and 75^th^ percentiles, and the whiskers indicate 95% confidence intervals through bootstrapping.

To assess the robustness of the AI models to various clinical factors, we evaluated their performance across centers, ultrasound systems, histological diagnoses, examiner confidence levels, patient age groups and years of examination. The F1 scores of the AI models and human examiners by centers are shown in Fig. 3a. The AI models consistently outperformed both expert and non-expert examiners, except for the Monza and Cagliari centers (Fig. 3a and Supplementary Table 3). We also examined model performance for different ultrasound systems and found that the AI models exhibited robust performance, matching or surpassing the performance of expert examiners, irrespective of the ultrasound manufacturer or system used (Fig. 3b and Supplementary Table 4). We assessed model performance for different histological diagnoses, as illustrated in Fig. 3c and detailed in Extended Data Table 2. Also here, the AI models exhibit superior performance compared to human expert and non-expert examiners, even in cases known to be challenging to classify, such as cystadeno(fibro)mas, solid lesions and mucinous intestinal borderline tumors. The only exception to this trend was serous borderline tumors. For a detailed visual comparison, we show the differences between the performance of the AI models and the human examiners, by centers, ultrasound systems and histological diagnoses, as forest plots in Supplementary Fig. 1.

**Extended Data Fig. 2:**
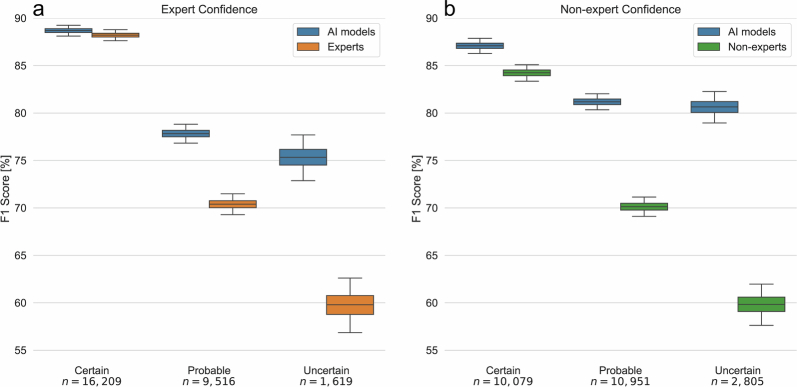
Performance of AI models and human examiners by level of confidence in assessment. F1 scores for (**a**) expert examiners and AI models and (**b**) non-expert examiners and AI models, partitioned by the examiner’s confidence in their assessment. For each level of confidence (certain, probable, uncertain), all assessments with the corresponding level of confidence were pooled. The box plots show the median and the 25^th^ and 75^th^ percentiles, and the whiskers indicate 95% confidence intervals through bootstrapping.

We explored the relationship between diagnostic performance and examiner confidence. When presented with a case, the examiner was asked to classify the lesion as benign or malignant and rate their confidence in the assessment as certain, probable, or uncertain. As expected, we noted a strong correlation between the examiners’ performance and their confidence, with a sharp decrease in performance when the examiners were uncertain. In contrast, the AI models demonstrated only a modest decline in performance in these challenging cases (Extended Data Fig. 2 and Supplementary Table 5).

**Extended Data Fig. 3:**
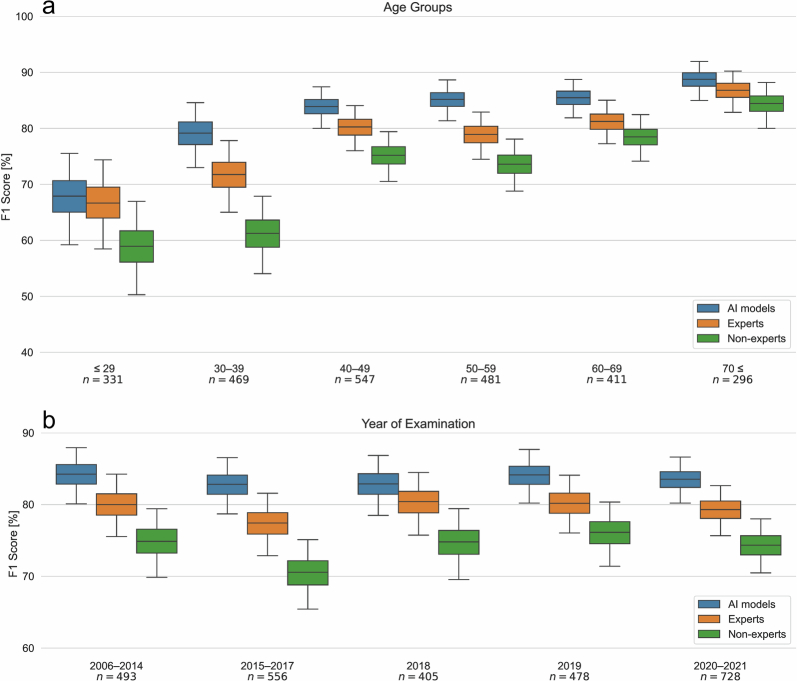
Subgroup analysis. Comparison of the AI models and expert and non-expert examiners, for different (**a**) age groups and (**b**) years of examination. The box plots show the median and the 25th and 75th percentiles, and the whiskers indicate 95% confidence intervals through bootstrapping. Information on patient age was missing for 125 patients.

We saw stable model performance independent of patient age (Extended Data Fig. 3a and Supplementary Table 6) and year of examination (Extended Data Fig. 3b and Supplementary Table 7), outperforming both expert and non-expert examiners across all subgroups.

Finally, for transparency, the performance of the AI model on the 644 excluded cases with known histological diagnoses from the Stockholm center is shown in Supplementary Table 8. The table shows that the performance of the AI model was similar or somewhat better on all metrics on these remaining 644 cases, compared to the 300 cases from the Stockholm center that were included in the main analysis.

### Training with specific histological diagnoses

Although our goal was to differentiate between benign and malignant lesions, the models were trained to discern ten different histological categories within the benign and malignant classes. This was done to leverage the richer information contained in the specific histological diagnoses.

To investigate the impact of diagnosis granularity on AI model performance, models were trained using binary labels and 18 specific histological diagnoses, besides the default setup with ten different histological categories. As seen in Supplementary Table 9, training with ten histological categories significantly improved model performance compared to training with binary labels (F1 83.50% versus 82.22%; Δ = 1.28 (95% CI, 0.14–2.47, *P* = 0.029)).

### Model calibration

**Extended Data Fig. 4:**
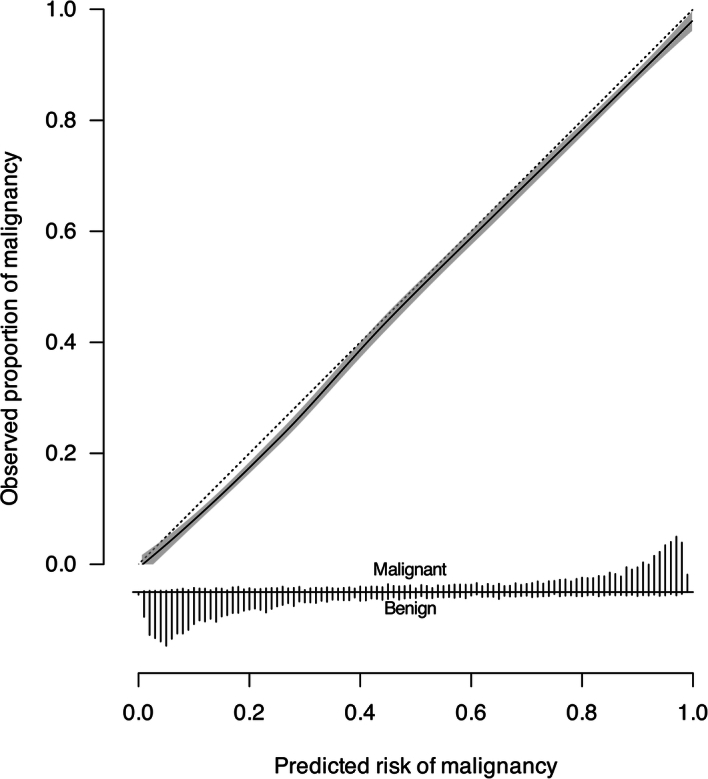
Calibration curve of AI models. A calibration curve of the AI models is shown in solid black with 95% confidence bands in gray, depicting the relationship between the predicted risk of malignancy and the actual observed proportion of malignancy. The dotted line represents the ideal scenario of perfect calibration, where the predicted risks precisely match the observed outcomes. The histograms at the bottom depict the distributions of predicted risks of malignancy, for malignant and benign tumors, above and below the horizontal line, respectively. The calibration curve and confidence bands are based on local regression (loess),24 and is based on 12,673 image-level predictions. While not depicted in this figure, a linear logistic calibration curve was also fitted, yielding an intercept of −0.19 (95% CI, −0.24–(−)0.14) and a slope of 1.00 (95% CI, 0.96–1.03), also indicating well-calibrated risk predictions.

For a model to be effectively integrated into clinical practice, high diagnostic accuracy is necessary but not sufficient; the model must also exhibit robust calibration. This aspect is particularly crucial for models intended for diagnostic support, rather than stand-alone systems, as it underpins the establishment of clinicians’ trust in the technology. To assess the calibration of our AI models, we utilized a calibration curve (Extended Data Fig. 4)^23,24^. The calibration curve of the AI models showed good correspondence between the predicted risk of malignancy and the actual observed proportion of malignancy, indicating well-calibrated predictions. This means the model confidence is strongly correlated with the likelihood of them making a correct prediction. In other words, the models tend to be confident only in cases where they are likely to make a correct diagnosis.

### Image cropping

**Extended Data Fig. 5:**
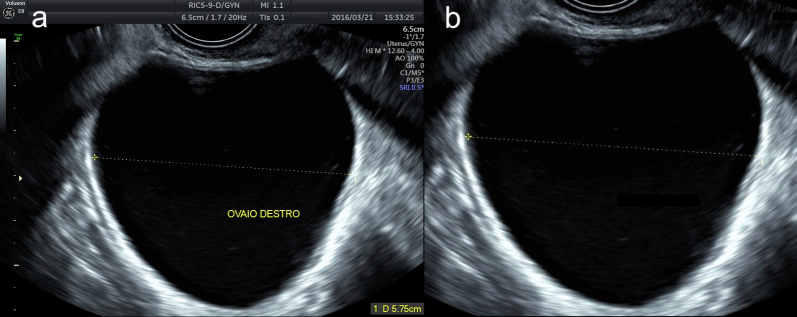
Image cropping and annotation. (**a**) An uncropped image, as provided by a participating center, and (**b**) the corresponding cropped image used for training and evaluation. Images were coarsely cropped, mainly by removing the outer borders and burnt-in scanner settings, and occasionally also excluding surrounding structures. Within the cropped images, artifacts such as text were blacked out by setting the pixel values to zero.

**Extended Data Fig. 6:**
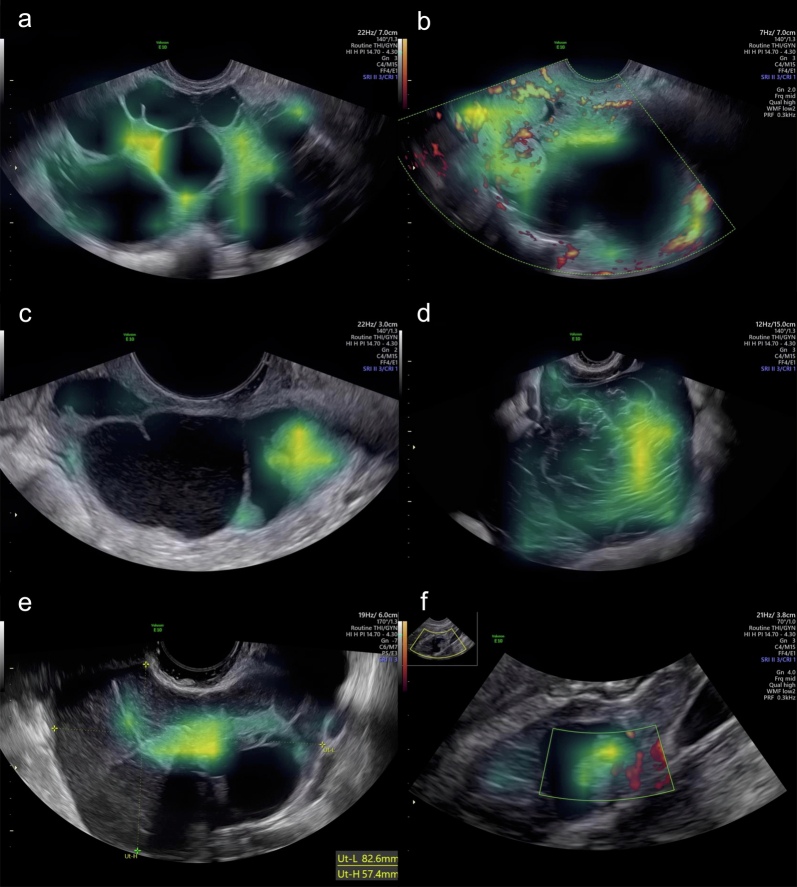
Saliency maps. Attention-based saliency maps from the AI models for a few uncropped images of a (**a**) serous cystadenoma, (**b**) tubal cancer, (**c**) urothelial cancer metastasis, (**d**) colorectal cancer metastasis and (**e–f**) serous borderline tumors. The attention maps demonstrate that the models focus on areas of clear diagnostic relevance, such as (**b**) vascularized and (**e**) irregular (**a**–**c**) solid components, (**d**) with densely packed locules and (**f**) papillary projection, while ignoring image artifacts, such as text, (**e**) calipers or (**f**) thumbnails.

Four gynecology residents were tasked with manually selecting a rectangular region of interest (ROI) in each image, whereby the lesion was centrally located and occupied most of the image. This task was performed in the data labeling platform SuperAnnotate. The involvement of gynecology residents aimed to avoid potential bias and dependence on advanced domain expertise not always present in routine clinical practice. We used images cropped to the annotated ROIs for both model training and evaluation. The residents also marked artifacts other than calipers, for example, text, inside the ROI for removal (Extended Data Fig. 5). This was done to reduce the risk of bias and to prevent the models from picking up on artifacts in the images during training that are not useful for the classification task. We explored the impact of image cropping and observed only a marginal decrease in model performance when applied to uncropped images (Supplementary Table 10), suggesting that cropping may not be a necessary step for achieving good model performance. Artifact removal at evaluation had minimal impact on model performance (Supplementary Table 10). In Extended Data Fig. 6, we show attention-based saliency maps for a few uncropped images, highlighting image locations that were relevant to the model’s predictions^25^. The figure demonstrates that the model does not focus on image artifacts, such as text, calipers and other annotations, when making a prediction, but rather on areas of clear diagnostic relevance. This provides further validation of the model’s ability to locate and prioritize clinically relevant features, enhancing its reliability and interpretability.

As an additional evaluation of the need for manual ROI selection, we evaluated the models on auto-cropped images. For this, we used the same leave-one-center-out cross-validation scheme as for the transformer-based classification models. For each center in turn, we trained an object detection model based on YOLO (version 8)^26^, for the task of predicting the ROI in an image. For the training of these models, we used the ROIs that had been manually annotated by four gynecology residents as earlier described. As seen in Supplementary Table 10, evaluation on auto-cropped images performed on par with evaluation on manually cropped images (without artifacts removed).

### Triage simulation

**Fig. 4:**
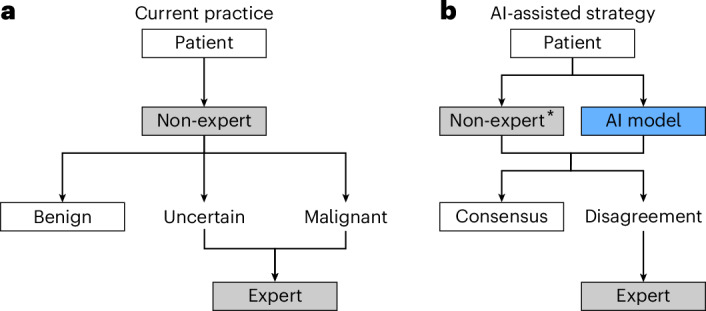
Current practice and proposed AI-assisted strategy for triage workflow. **a**, In the current practice, a non-expert examiner makes an initial assessment, and patients with an uncertain diagnosis or presumed malignancy are referred to an expert. Additionally, with gynecologists in training (residents), most newly detected lesions are referred to an expert examiner, independently of the finding. **b**, In our proposed AI-assisted triage strategy, the AI model and a non-expert examiner each make an initial assessment, and then an expert examiner makes the final decision in cases of disagreement. *The proposed AI-assisted strategy can also be used with an expert as the initial examiner.

The clinical expertise and certainty of the examiner, as well as the availability for review by an expert examiner or magnetic resonance imaging (MRI), determine the current clinical triage routine. With gynecologists in training (residents), most newly detected lesions are referred for a second opinion or expert ultrasound assessment, whereas with more experienced gynecologists, only cases with an uncertain diagnosis or presumed malignancy are referred for second opinion or expert ultrasound assessment, or MRI in selected cases (Fig. 4a). AI-driven diagnostic support has the potential to alleviate the shortage of expert examiners and improve patient outcomes by optimizing clinical workflow. We proposed to integrate AI-assistance into the triage routine as a second reader. The AI model and a human examiner (expert or non-expert) each make an initial assessment, and then an expert examiner makes the final decision in cases of disagreement (Fig. 4b).

Leveraging the OMLC-RS dataset, we simulated and assessed how this modified clinical workflow affects diagnostic accuracy and human resource demands (Table 2). As a second reader, the AI model improved diagnostic performance in comparison to the current triage routine for non-expert examiners (F1 82.70% versus 77.16%; Δ = 5.54 (95% CI, 4.11–6.98, *P* < 0.0001)). This AI-assisted strategy both elevated diagnostic accuracy and reduced human resource demands (that is, the number of examinations needed to make a management decision), from the current practice of 1.52 (non-expert examiners) to 1.19, a 63% reduction in referrals to experts. The reduction would be even greater among gynecologists in training (residents), where most newly detected lesions are referred to an expert examiner independently of the finding. A similar trend was found for expert examiners, where the AI model as a second reader improved the F1 score from 79.50% to 83.56% (Δ = 4.05 (95% CI, 2.99–5.42, *P* < 0.0001)) while incurring only a marginal increase in human resource demands, from 1.00 to 1.15 (Table 2).

### Conservatively managed patients

The main evaluation of the AI models and their comparison with human examiners were limited to patients having a post-surgical histological diagnosis. However, the prevalence of various specific benign tumor types in this group may differ from those found among patients managed conservatively with ultrasound follow-up. As this could affect the transferability of our findings, we separately evaluated the AI models on images from 233 patients from the Stockholm center who had been managed conservatively with ultrasound follow-up, yielding a specificity of 92.70% (*n* = 216/233) (Jeffrey’s Bayesian 95% CI, 88.83–95.53)^27^, whereas the sensitivity is undefined, as all lesions were benign.

## Discussion

To the best of our knowledge, this is the first comprehensive study that systematically explores and validates the potential of AI models in multiple international external centers for distinguishing between benign and malignant ovarian lesions in ultrasound images, with comparison to human examiners. Our findings demonstrate the strong generalization capability of transformer-based neural network models that performed better than every expert and non-expert examiner. This trend was consistent for different ultrasound systems, histological diagnoses and, most importantly, unseen patient populations from centers the models had not been trained on.

Our retrospective triage simulation demonstrated the potential of AI-driven support in enhancing diagnostic accuracy while simultaneously substantially reducing the need for second opinion and referrals to experts. This finding is especially vital given the scarcity of expert examiners, underlining AI’s potential for advancing equitable access to high-quality diagnostic services. In contrast to human examiners, the AI models maintained high performance even in cases where human examiners were uncertain. This suggests that AI-driven diagnostic support may have a particularly important role in cases that are difficult to classify by human examiners.

The calibration curve showed that the AI models are well calibrated (Extended Data Fig. 4). We believe this to be a result of our model architecture, as transformer-based models have been shown to be better calibrated compared to CNNs for natural images^28^. As ultrasound images have different properties compared to natural images, we created a calibration curve using CNN-based models for comparison (Supplementary Fig. 2). The results are in line with Minderer et al.^28^, suggesting that the favorable calibration properties of transformer architectures may extend to ultrasound images. Furthermore, the use of focal loss (Methods) during training is known to improve model calibration compared to the standard cross-entropy loss^29,30^.

Surprisingly, we saw only a marginal decline in model performance when evaluated on uncropped images, despite the models never encountering uncropped images during training (Supplementary Table 10). Furthermore, evaluation on auto-cropped images performed on par with evaluation on manually cropped images, which suggests the utility of AI in simplifying clinical workflow by eliminating the need for manual ROI indication. Regarding explainability, various methods based on saliency maps and feature similarity have been proposed^31,32^. We visually inspected attention-based saliency maps (Extended Data Fig. 6), which demonstrated that the model does not focus on spurious image artifacts but rather on areas of clear diagnostic relevance.

The main strength of our study lies in the diverse OMLC-RS dataset and the rigorous evaluation. By ensuring that no model was ever trained and tested on cases from the same center, we avoided overly optimistic results commonly encountered in retrospective studies^18^. To illustrate this, we conducted a separate experiment where a model was evaluated using data from a center included during training, observing inflated results (Supplementary Table 11).

The inclusion of a substantial cohort of both expert and non-expert examiners mirrored the diversity inherent in clinical practice. This enabled a comprehensive analysis comparing the performance of AI models and human examiners.

Although our study upholds rigorous standards, we acknowledge its retrospective nature as a limiting factor. The human examiners assessed cases solely based on ultrasound images, which may underestimate their performance in a clinical setting, as additional clinical information may lead to enhanced diagnostic performance. However, clinical variables could also be incorporated into AI models. Furthermore, the level of experience and expertise among the human examiners in this study, especially the expert examiners, most likely exceeds that of the average examiner in the corresponding examiner category, and therefore, we may underestimate the difference in diagnostic performance between the AI models and the examiners. The main comparison between the AI models and human examiners was limited to patients with a post-surgical histological diagnosis. This limitation may affect the transferability of our findings to patients managed conservatively with ultrasound follow-up. Consequently, further studies are needed to validate our models in conservatively managed patient populations. However, on a separate set of 233 conservatively managed patients, the AI model achieved a specificity of 92.70% (95% CI, 88.83–95.53). Although we did not compare against human examiners in this cohort, and despite these patients all being from the same external center, we find the results promising as it points to the potential applicability and reliability of the AI models also in this setting. Our models outperformed both expert and non-expert examiners on all prevalence independent metrics, that is, sensitivity, specificity, DOR and Youden’s J statistic. Nevertheless, as is the case for all metrics, also prevalence independent metrics may be affected by the spectrum of various specific tumor types and severity^33–35^, which in turn depend on the clinical setting. As most cases were referral scans, future studies should evaluate the models’ effectiveness in settings with lower prevalence, outside of ultrasound referral centers. As another limitation, most patients in our study were scanned by an experienced examiner at their center of inclusion. This retrospective study used images originally acquired for archival in patients’ medical records, not for image analysis, likely resulting in suboptimal image quality. Regardless, further studies are needed to evaluate the models’ performance on images obtained by less experienced examiners specifically for AI evaluation.

In a recent systematic review by Koch et al.^36^, only three studies were identified that utilized external validation to assess automated computer-aided diagnostic systems for ovarian cancer detection based on ultrasound imaging. These studies were all retrospective and only one, conducted by Gao et al. using a CNN model^10^, used a reasonably sized test set (the remaining studies included only 15 or fewer benign cases). However, in the study by Gao et al., their model’s performance was externally compared to human examiners in only a single center, including a limited sample size of 335 cases (268 benign and 67 malignant)^10^. Relying on a single external center for evaluation of robustness and generalizability may yield unreliable conclusions. Their study was further listed by Koch et al. as containing high risk of bias, as very little of their analysis process is described^36^. A key differentiator of our study is the size and diversity of our dataset, as well as our comprehensive evaluation. We report results on 2,660 cases (1,575 benign and 1,085 malignant) from 19 external centers, with comparison to a large cohort of human examiners (33 non-experts and 33 experts). We demonstrate robustness across many external centers (Fig. 3a and Supplementary Table 3), various ultrasound systems (Fig. 3b and Supplementary Table 4), histological diagnoses (Fig. 3c and Extended Data Table 2), patient age groups (Extended Data Fig. 3a and Supplementary Table 6), years of examination (Extended Data Fig. 3b and Supplementary Table 7) and perceived case difficulty based on examiners’ confidence in their assessments (Extended Data Fig. 2 and Supplementary Table 5). Furthermore, we show that our models are well calibrated (Extended Data Fig. 4), whereas Gao et al. reported calibration curves indicative of a highly overconfident model with a systematic underestimation of the risk of malignancy^10,37^. Our models significantly outperformed both expert and non-expert examiners on all evaluated metrics. Meanwhile, the model by Gao et al. had a significantly lower sensitivity compared to that of their mean examiner (40.3% versus 55.5%), despite their cohort of examiners being relatively inexperienced, with a diagnostic performance substantially lower than what has been reported in other studies^10,38^.

Besides the size and diversity of our dataset, we also attribute the robust performance and generalization capabilities to our model architecture and training methodology. Our complementary experiments showed that CNN models yield marginally lower performance and worse calibration compared to the transformer-based model architecture that we adopted (Supplementary Table 12 and Supplementary Fig. 2), as also found by Matsoukas et al.^21^. In addition, the inclusion of specific histological diagnoses during training significantly improved model performance (Supplementary Table 9).

In conclusion, our study demonstrates the potential of AI models in improving the accuracy and efficiency of ovarian cancer diagnosis. Our models demonstrated robust generalization and significantly outperformed both expert and non-expert examiners on all evaluated metrics. The additional triage simulation in our study offered valuable insights into the practical potential of AI model integration into a clinical diagnostic routine. Although further prospective and randomized studies are needed to validate the clinical benefit and diagnostic performance of the AI models, and to investigate their influence on examiners’ management decisions, our study offers insights into the applicability of AI-driven diagnostic support systems in the field of ovarian cancer detection. The models’ consistent superiority to human assessment and robust performance under comprehensive evaluation indicates that they are ready for prospective clinical implementation studies, bringing us closer to the adoption of AI-assisted diagnostics in clinical settings.

## Methods

### Data acquisition

In this international multicenter retrospective study, we included transvaginal and transabdominal ultrasound images from patients with an ovarian lesion, examined between 2006 and 2021 at 20 secondary or tertiary referral centers for gynecological ultrasound in eight countries. The images were acquired by examiners with varying levels of training and experience, using 21 different commercial ultrasound systems from nine manufacturers, primarily GE (91.8%), followed by Samsung (4.8%), Philips (1.4%) and Mindray (1.2%) (Supplementary Table 13). Participating centers were requested to provide images of at least 50 consecutive malignant cases and at least 50 benign cases, examined just before or after each malignant case, to ensure a similar temporal distribution between classes and avoid bias from potential variations in diagnostic practices or equipment over time. This enrichment strategy was designed to ensure an adequate representation of malignant cases, thereby more effectively capturing rare pathologies while minimizing potential biases^17^. The inclusion of images for a given patient was limited to the side of the lesion, and in cases of bilateral lesions, the side of the dominant lesion (that is, that with the most complex ultrasound morphology) was included. Anonymized images were submitted in JPEG format. Data transfer agreements were signed between the host institution, Karolinska Institute, and each of the participating centers. The study was preregistered at 10.1186/ISRCTN51927471, approved by the Swedish Ethics Review Authority (Dnr 2020-06919) and conducted in accordance with the Declaration of Helsinki. Informed consent had been obtained from all patients for the use of their data for research purposes.

After excluding 4.8% (*n* = 183/3,840) of the cases (91 benign and 92 malignant) due to inadequate image quality (for example, lesions that could not be identified, lesions with blurred margins and lesions that were only partially visible), 17,119 ultrasound images (10,626 grayscale and 6,493 Doppler) representing 3,652 cases remained for analysis (Extended Data Fig. 1). Out of these cases, 3,419 were patients who had undergone surgery, including histological assessment, within 120 days of their ultrasound examination. The remaining 233 patients had been managed conservatively with ultrasound follow-up until the resolution of the lesion, or for at least three years without a malignant diagnosis, and were thus regarded as benign. The median number of images per case was 4 (interquartile range (IQR): 3–6). A breakdown of the diagnoses is shown in Table 1 and by center in Supplementary Fig. 3. Specific histological diagnoses are provided in Supplementary Table 14, a detailed summary of the data by centers can be found in Extended Data Table 3, and by centers separately for benign and malignant cases in Supplementary Table 15.

### Human examiner review

To ensure a thorough evaluation, we collected the assessments made by 66 human examiners, comprising 33 ultrasound experts and 33 non-experts, recruited at the participating centers. To establish a competitive baseline and ensure the validity of our results, expert examiners were recruited based on their extensive expertise in gynecological ultrasound imaging for the assessment of ovarian lesions. For our study, an ‘expert’ examiner was defined as a physician who performs second or third opinion gynecological ultrasound imaging, and who has at least 5 years’ experience or annually assesses at least 200 patients with a persistent ovarian lesion. Among the experts, the median experience in gynecological ultrasound imaging was 17 years (IQR: 10–27 years), with a median of 10 years as second or third opinion (IQR: 5–17 years). Most experts (91%, *n* = 30/33) were affiliated with a gynecologic oncology referral center, 61% (*n* = 20/33) performed over 1,500 gynecological ultrasound scans annually, and 64% (*n* = 21/33) reported seeing more than 200 patients with a persistent ovarian lesion each year. To strive for a fair evaluation, we did not train the ‘non-expert’ examiners beyond providing them with instructions for the task. The specific prior training and certification varied among examiners, as they were included from centers in eight different countries. However, all non-expert examiners were certified physicians, actively practicing gynecological ultrasound imaging. They had a median experience of 5 years (IQR: 3–6 years) and 52% (*n* = 17/33) were affiliated with a gynecologic oncology referral center. Furthermore, 24% (*n* = 8/33) of non-experts served as second or third opinion referrals, however, not meeting the criteria for an ‘expert’ examiner determined in this study. When presented with a case, the examiner was asked to classify the lesion as benign or malignant using pattern recognition (that is, subjective ultrasound assessment)^40^, and rate their confidence in the assessment as certain, probable, or uncertain. To prevent bias from previously seen cases, none of the examiners were asked to review cases originating from their own centers.

A total of 2,660 cases (1,575 benign and 1,085 malignant) were assessed by at least 7 expert (median: 10, IQR: 9–11) and 6 non-expert (median: 9, IQR: 8–10) examiners, with a total of 51,179 assessments. The median number of cases assessed by each expert and non-expert examiner was 696 (IQR: 628–886) and 610 (IQR: 583–655), respectively. One center (Olbia) was excluded from the review due to its limited sample size (*n* = 57) and its small number of malignant cases (*n* = 8). Additionally, 58 cases from three centers (Cagliari, Trieste and Pamplona) were excluded from our main analysis as these had not been included in compliance with our criterion on the temporal distribution of examination dates. After excluding 233 patients managed conservatively with ultrasound follow-up, we selected 300 cases (150 benign and 150 malignant) from the Stockholm center with known histological diagnoses for inclusion in the human review. We selected the most recent 150 consecutive malignant cases, followed by one benign case examined just before or after each malignant case. The remaining 644 cases from the Stockholm center were excluded to have a test set of comparable size to those of the other centers and to utilize our reviewer resources efficiently. The excluded cases (*n* = 57) from the Olbia center were used as supplementary training data for all models. The 877 cases excluded from the Stockholm center (233 conservatively managed and 644 with post-surgical histological diagnosis) were also used as supplementary training data; however, only when the Stockholm center was not the held-out test set.

### Model training

The OMLC-RS dataset was used to train a series of 19 transformer-based neural network models, each using DeiT architecture initialized with ImageNet pretraining^20,41^. We applied a leave-one-center-out cross-validation scheme, where iteratively each center in turn was isolated as the test set and the model was given the cases from the remaining centers for training. More specifically, in each iteration, the cases from the remaining centers were randomly split into a training (90%) and a validation (10%) set, with the validation set used for selection of the learning rate. A caveat to the procedure is that the random split was constrained such that the validation set had an equal number of malignant and benign cases. When we say that a case was used for training, we mean that it was included in either the training set or validation set.

Although our goal was to differentiate between benign and malignant lesions, the models were trained to discern ten different histological categories within the benign and malignant classes (Supplementary Table 14), which was done to leverage the richer information contained in the specific histological diagnoses. We trained the models using the multiclass focal loss^42^, which encourages the model to assign greater importance to often misclassified examples compared to the standard cross-entropy loss^30^.

### Image pre-processing

Before training, images were cropped to the regions of interest, unless otherwise stated. The cropped images were zero-padded to square shape and resized to 256 × 256 x 3 pixels. The mean and standard deviation of the pixels for the images in the dataset were then computed for each color channel for later use.

For each training epoch, images were loaded from disk and randomly cropped to 224 × 224 × 3 pixels. The RandAugment method was used for data augmentation^43^, with default hyperparameters, five sequential random transformations and color-related transformations removed. Thereafter, the image pixels were normalized to zero mean and unit variance, using the precomputed pixel statistics.

### Additional training details

Transformer-based models originate from the field of natural language processing^44^, an area that has seen immense progress in recent years with the advent of large language models^45^. Transformer-based models have been adapted and increasingly utilized also for imaging tasks. Within the ultrasound domain, these models were first used by Gheflati et al. in 2022 for the classification of breast lesions^46^. In our study, we used the DeiT-S (DeiT small) architecture^20^, with transfer learning from model weights initialized with ImageNet pretraining^41^. Transfer learning from ImageNet has become a standard approach and has been shown to improve performance in medical imaging tasks^21^. In our preliminary investigation, we also tried the larger model version, DeiT-B (DeiT base); however, as there were no noticeable improvements, we used the smaller DeiT-S architecture for computational efficiency. The linear projection layer on top of the final hidden state of the class token was replaced by a new linear projection layer with ten nodes, that is, with the same dimensionality as the number of classes. The AdamW optimizer was used^47^, with default hyperparameters, except for the learning rate. For each experiment, four different learning rates (10^−3^, 10^−4^, 5 × 10^−5^ and 10^−5^) were tried, each with a linear warm-up for 500 training steps and a batch size of 128 images. When the performance on the validation set reached a plateau, the learning rate was reduced. This reduction was made twice, each time by a factor of 0.1.

At the end of training, the model with the best performance on the validation set was selected, based on the case-wise binary classification performance in terms of the area under the ROC curve (AUC). An exponential moving average of the model weights from each training epoch was computed using a decay factor of 0.99. These model weights were later used for model evaluation.

### Model inference

After training, the multi-class neural network models provided probability estimates for each of the ten histological categories within the benign and malignant classes (Supplementary Table 14). Because our goal was to differentiate between benign and malignant lesions, we computed the risk of malignancy for an image by summing up the probabilities for the five malignant classes, in a manner similar to Esteva et al.^48^. The malignancy score for a case was then computed as the average of the malignancy scores of its images. A case was considered malignant if its malignancy score exceeded a given cut-off point. Unless otherwise stated, we used the default cut-off point of 0.5.

### Evaluation procedure

To avoid overly optimistic results commonly seen in medical machine learning^18^, we conducted a rigorous assessment of the diagnostic performance of our models via separate test sets, each containing only data from the center withheld during training. We compared the predictions of the models and the expert and non-expert examiners with histological diagnosis from surgery. We used the F1 score as the primary metric as it provides a balance between precision and recall, and which unlike the AUC can be computed in a straightforward and unbiased way also for human examiners. The F1 score is the harmonic mean of the precision (that is, positive predictive value) and the recall (that is, sensitivity):

$${\mathrm{F}}1=2\frac{{\mathrm{PPV}}\times {\mathrm{sensitivity}}}{{\mathrm{PPV}}+{\mathrm{sensitivity}}}$$

Metrics were calculated at the case level, as opposed to image-wise. In addition to the F1 score, we also report accuracy, sensitivity, specificity, Cohen’s kappa coefficient, MCC, DOR and Youden’s J statistic, as well as the AUC and Brier score for the models. The primary evaluation in our study compared the performance of the AI models with each individual examiner’s assessments on matched case sets. When calculating the diagnostic performance of the models, we identified the originating center for each case and used the model that had not been exposed to cases from that center during training.

### Statistical analysis

To compare the diagnostic performance of the AI models with that of expert and non-expert examiners, we applied two-sided non-parametric Wilcoxon signed-rank tests (Supplementary Table 1)^49^, performed in JASP (version 0.18.3).

We evaluated the robustness of the AI models by examining performance variations across different centers, ultrasound systems, histological diagnoses, examiner confidence levels, patient age groups and years of examination. Rather than statistical tests, box plots and nonparametric confidence intervals were provided. Confidence intervals were estimated from bootstrapping using the percentile method^50^, as direct parametric calculation of the confidence intervals was not possible for the human examiners.

To ensure unbiased examiner representation, we used a sampling strategy where each examiner was selected with a probability inversely proportional to their number of cases assessed. This strategy was consistently applied also in our triage simulation.

Additionally, we assessed the sensitivity-specificity trade-off by presenting an ROC curve for the AI models, accompanied by 95% confidence bands. The confidence bands were constructed from the 2.5th and 97.5th percentiles of sensitivity values, at each level of specificity, from bootstrapped ROC curves. We also depicted 95% confidence regions for the mean diagnostic performance of expert and non-expert examiners. To account for the negative correlation between sensitivity and specificity, we applied a bivariate random-effects model^39^, implemented in SAS (version 9.04). The calibration plots were constructed using R (version 4.3.3).

All other analyses, including bootstrapping and triage simulations, were conducted using Python (version 3.8.13) with the pandas library (version 2.0.1). A significance level of 0.05 was used for all statistical tests.

Our initial power analysis, which was based on our plan to compare the AI models with the initial assessments of the ultrasound examiners who generated the images, resulted in a required sample size of 1,600 cases. To account for potential dropout, we initially requested a minimum of 100 cases from each of the 20 participating centers. The inclusion process exceeded our expectations, resulting in a total of 3,652 cases. However, as the examiners’ initial assessments had not been systematically documented for most centers, we adjusted our evaluation strategy as detailed in the ‘Human examiner review’ section.

### Reporting summary

Further information on research design is available in the Nature Portfolio Reporting Summary linked to this article.

## Online content

Any methods, additional references, Nature Portfolio reporting summaries, source data, extended data, supplementary information, acknowledgements, peer review information; details of author contributions and competing interests; and statements of data and code availability are available at 10.1038/s41591-024-03329-4.

## Supplementary information

Supplementary Information Supplementary Figs. 1–3 and Tables 1–15.

Reporting Summary

## Figures and Tables

**Extended Data Table 1:**
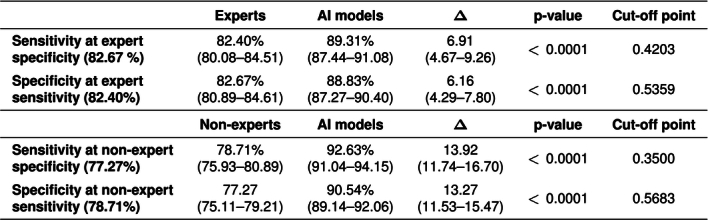
Sensitivity/specificity of AI models and human examiners at matching specificity/sensitivity

Data are % (95% CI) or percentage points (95% CI). The p-values are based on two-sided non-parametric confidence interval tests and indicate a statistically significant difference between the diagnostic performance of the AI models and the human examiners, in all cases.

**Extended Data Table 2:**
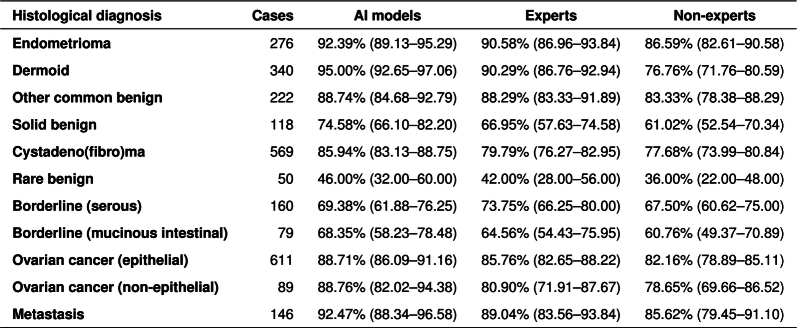
Sensitivity of AI models and human examiners partitioned by histological diagnosis

Data in parentheses are 95% CIs.

**Extended Data Table 3:**
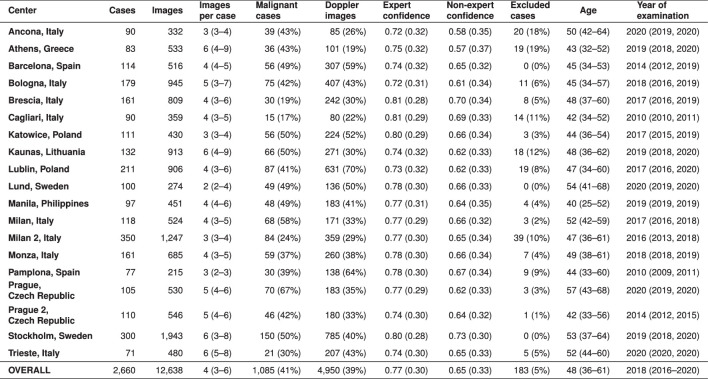
Center-wise summary of test dataset characteristics

The confidence represents the examiners’ average confidence in their assessments. The raw confidence scores were mapped from uncertain, probable, and certain, to 0, 0.5 and 1, respectively. Data are n, n (%), median (IQR) or mean (SD). Excluded cases refers to cases excluded due to inadequate image quality. Additional cases from three centers (Cagliari, Trieste and Pamplona) were excluded from our main analysis due to non-compliance with our criterion on the temporal distribution of examination dates.

## Data Availability

Because the examiners did not review cases from their own centers, their assessments will not be made publicly available or shared, as this would expose the identities of the individual examiners. The image data used in this study are not publicly available due to privacy concerns and study-specific data sharing agreements with multiple medical institutions across several countries that prohibit further sharing. However, researchers interested in conducting analyses or external model validation can submit their code as a dockerized container. We will run this code on our secure servers and provide the results back to the researchers without sharing any raw data. To initiate a request, please contact the corresponding author with a complete study protocol, including a clear research purpose and a detailed description of the proposed analysis. Detailed instructions will be provided upon approval of the request. Requests from academic investigators without relevant conflicts of interest and intended for noncommercial use will be evaluated within 2 months based on institutional policies, scientific merit and the availability of resources required to process the request. All other data supporting the findings of this study are available within the article and its Supplementary Information files.

## Code availability

The code that supports the findings of this study is under pending patent protection (European patent application 23220765.4) and cannot be publicly released. The code was offered to the editors and peer reviewers at the time of submission for the purposes of evaluating the manuscript. The technical details of the model training are described in sufficient detail in Methods to allow replication of our experiments using an open-source deep-learning framework, such as PyTorch or TensorFlow. The ImageNet pretrained models are freely available online.
