# Supplementary material for: International multicenter validation of AI-driven ultrasound detection of ovarian cancer
Source: Nat Med. 2025 Jan 2;31(1):189–96. doi: 10.1038/s41591-024-03329-4 (PMC11750711; doi:10.1038/s41591-024-03329-4)
Supplement: Supplementary file 1 — Supplementary Figs. 1–3 and Tables 1–15. [file 41591_2024_3329_MOESM1_ESM.pdf]

nature medicine

Supplementary information https://doi.org/10.1038/s41591-024-03329-4

International multicenter validation of AI-driven ultrasound detection of ovarian cancer

In the format provided by the authors and unedited

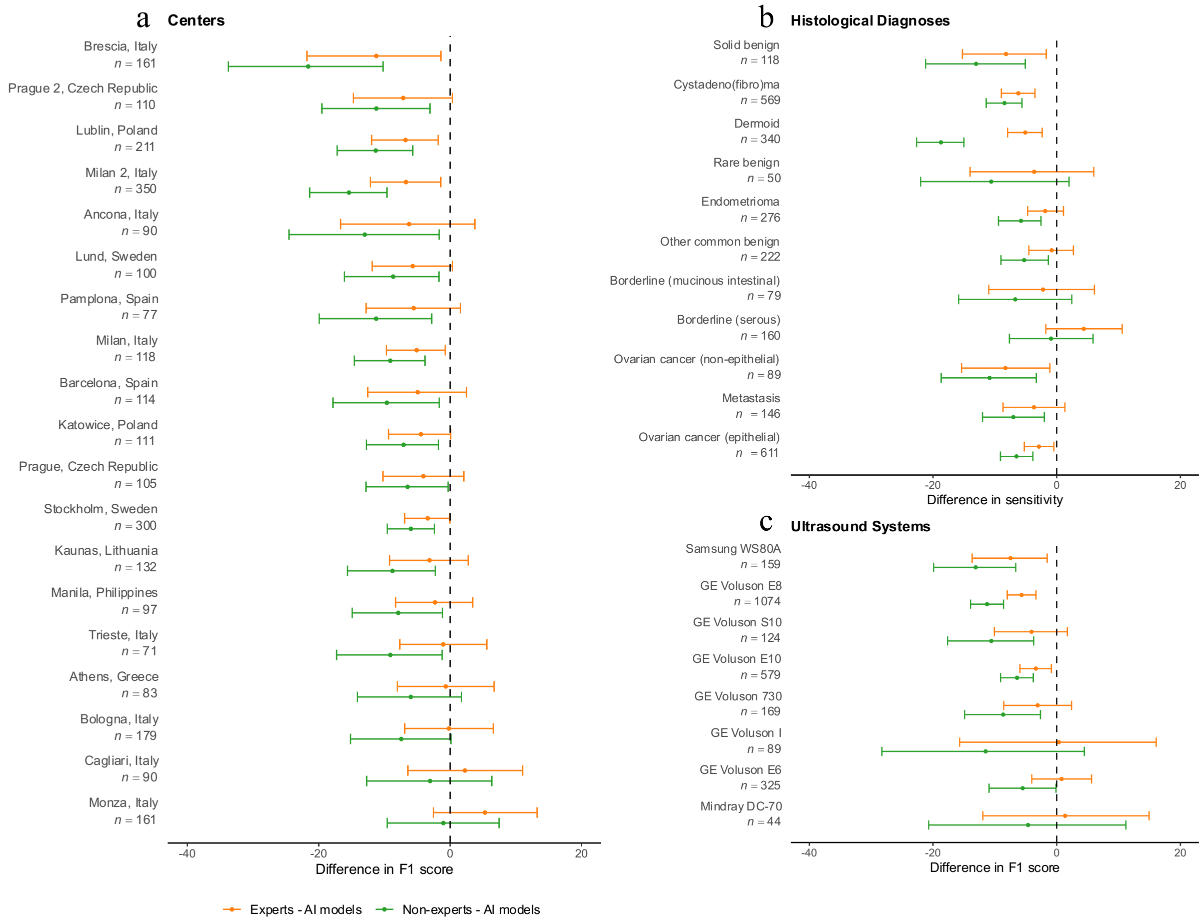

Supplementary Fig. 1 | Difference in performance between AI models and human examiners on various sub-groups. Differences in F1 scores between the AI models and the human examiners, by centers (**a**), ultrasound systems (**b**) and histological diagnoses (**c**). Error bars are 95% CIs through bootstrapping.

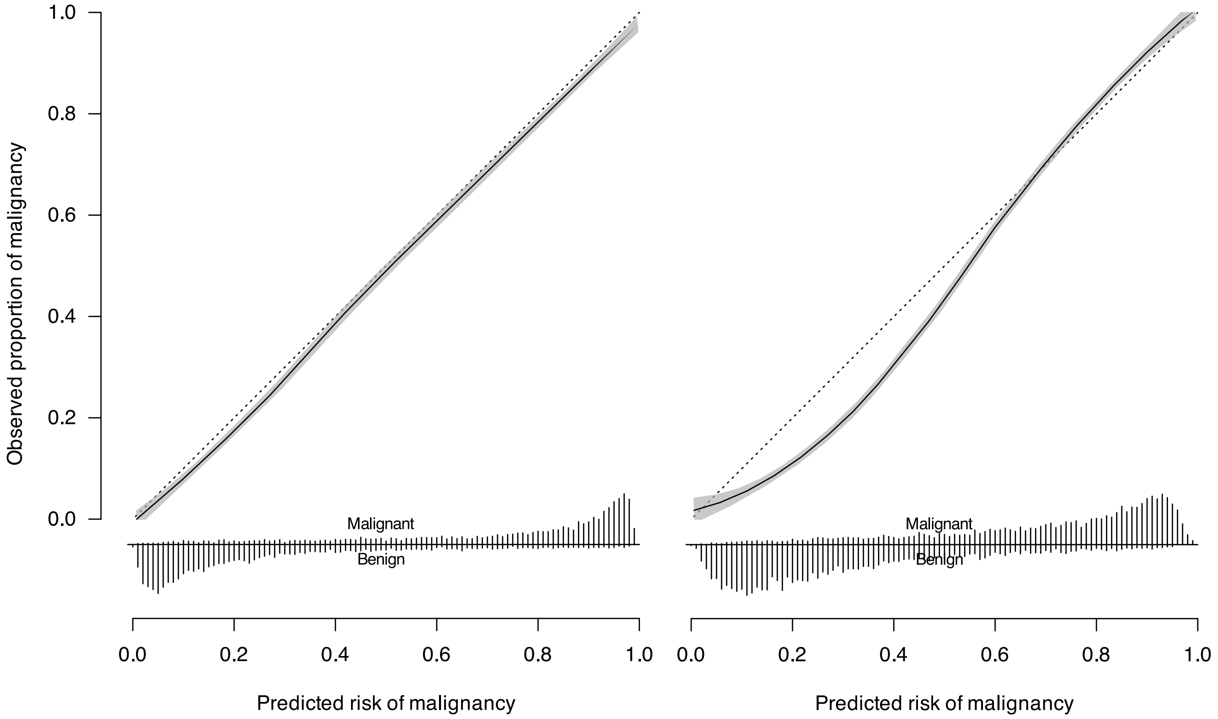

Supplementary Fig. 2 | Calibration curves of the transformer and CNN models. Calibration curves of (**a**) our transformer-based models (DeiT) and (**b**) convolutional neural network (CNN)-based models (ConvNeXt), respectively. The calibration curves are shown in solid black with 95% confidence bands in gray, depicting the relationship between the predicted risk of malignancy and the actual observed proportion of malignancy. The dotted lines represent the ideal scenario of perfect calibration, where the predicted risks precisely match the observed outcomes. The histograms at the bottom depict the distributions of predicted risks of malignancy, for malignant and benign tumors, above and below the horizontal line, respectively. The calibration curves and confidence bands are based on local regression (loess)[1], and is based on 12,673 image-level predictions. While not depicted in the figure, a linear logistic calibration curve was also fitted for each of the models, yielding (**a**) an intercept of -0.19 (95% CI, -0.24–(-)0.14) and a slope of 1.00 (95% CI, 0.96–1.03) for our transformer-based models, and (**b**) an intercept of -0.24 (95% CI, -0.29–(-)0.20) and a slope of 1.27 (95% CI, 1.23–1.32) for the CNN-based models.

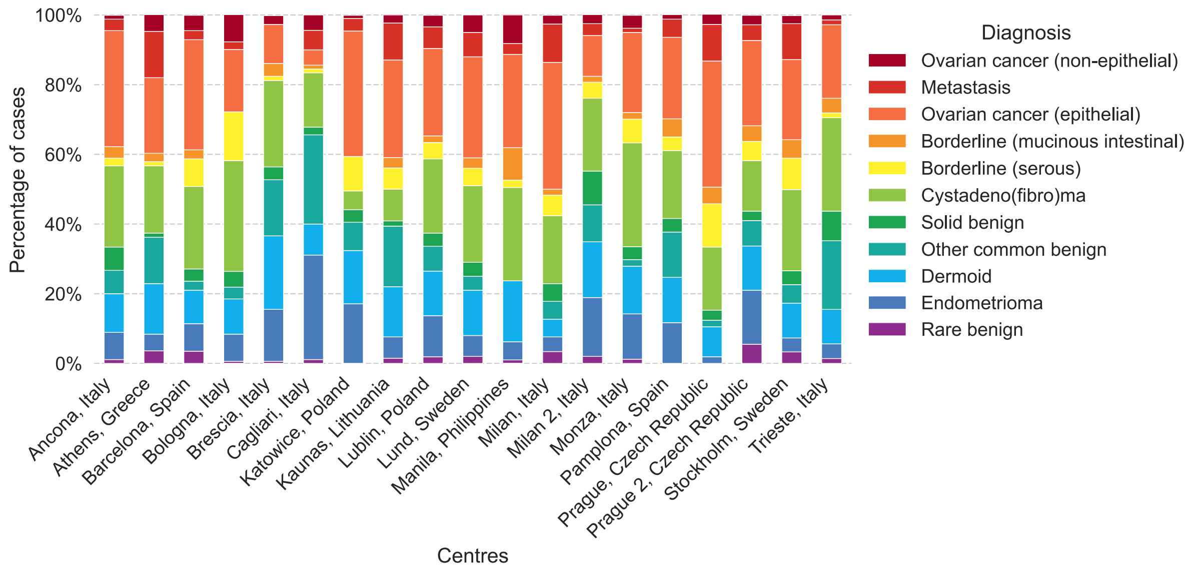

Supplementary Fig. 3 | Breakdown of histological diagnoses by center

Supplementary Table 1 | Hypothesis testing: AI models vs. expert and non-expert examiners

	W	z	p	Location parameter (Hodges-Lehmann estimate)	Effect size (Rank-biserial correlation)
**AI models vs. expert examiners**	561	5.012	2.328×10^{-10}	3.984	1.000
**AI models vs. non-expert examiners**	561	5.012	2.328×10^{-10}	8.979	1.000

Results of two-sided non-parametric Wilcoxon signed-rank tests comparing the diagnostic performance of the AI models with that of expert and non-expert examiners.

Supplementary Table 2 | Performance of examiners and AI models on matching case sets

Examiner	Cases	F1 score		Sensitivity		Specificity		Accuracy		Kappa		MCC	
		Examiner	AI models	Examiner	AI models	Examiner	AI models	Examiner	AI models	Examiner	AI models	Examiner	AI models
**Expert 1**	932	85.04%	85.16%	83.65%	84.47%	91.50%	90.97%	88.41%	88.41%	0.756	0.757	0.756	0.757
**Expert 2**	676	84.37%	85.30%	81.75%	85.77%	91.79%	89.55%	87.72%	88.02%	0.743	0.752	0.744	0.752
**Expert 3**	2,499	82.26%	83.67%	86.16%	85.21%	82.96%	86.50%	84.31%	85.95%	0.683	0.714	0.685	0.714
**Expert 4**	971	82.24%	83.27%	90.72%	85.31%	80.10%	86.96%	84.35%	86.30%	0.685	0.717	0.694	0.717
**Expert 5**	615	82.20%	84.05%	88.19%	85.04%	81.44%	87.81%	84.23%	86.67%	0.682	0.726	0.687	0.726
**Expert 6**	689	81.84%	86.42%	77.54%	88.77%	92.01%	88.86%	86.21%	88.82%	0.708	0.769	0.711	0.770
**Expert 7**	765	81.14%	84.75%	76.42%	86.27%	90.70%	86.51%	84.44%	86.41%	0.680	0.725	0.684	0.725
**Expert 8**	611	81.02%	86.27%	74.80%	87.80%	93.00%	88.80%	85.43%	88.38%	0.693	0.762	0.700	0.762
**Expert 9**	793	81.00%	86.06%	80.75%	87.27%	87.26%	89.38%	84.62%	88.52%	0.681	0.763	0.681	0.763
**Expert 10**	679	80.80%	84.43%	84.67%	86.21%	84.45%	88.76%	84.54%	87.78%	0.680	0.744	0.681	0.744
**Expert 11**	696	80.80%	82.48%	80.07%	84.42%	88.10%	86.67%	84.91%	85.78%	0.685	0.705	0.684	0.706
**Expert 12**	606	80.70%	83.46%	79.01%	82.82%	87.21%	88.08%	83.66%	85.81%	0.666	0.710	0.666	0.710
**Expert 13**	606	80.63%	84.73%	78.63%	84.73%	87.50%	88.37%	83.66%	86.80%	0.665	0.731	0.666	0.731
**Expert 14**	598	80.54%	82.54%	84.15%	84.55%	82.67%	85.80%	83.28%	85.28%	0.659	0.698	0.661	0.699
**Expert 15**	693	80.45%	83.79%	85.27%	83.22%	80.55%	88.78%	82.54%	86.44%	0.648	0.721	0.651	0.721
**Expert 16**	870	80.43%	83.12%	84.27%	83.71%	82.49%	87.74%	83.22%	86.09%	0.658	0.713	0.660	0.713
**Expert 17**	1,313	80.10%	82.34%	87.93%	83.52%	79.14%	87.23%	82.64%	85.76%	0.649	0.704	0.657	0.704
**Expert 18**	644	80.07%	84.62%	88.76%	86.52%	76.66%	87.27%	81.68%	86.96%	0.634	0.733	0.645	0.734
**Expert 19**	738	79.87%	83.73%	83.33%	85.76%	83.78%	87.78%	83.60%	86.99%	0.661	0.729	0.662	0.730
**Expert 20**	1,428	79.65%	84.08%	88.02%	85.24%	77.70%	88.15%	81.86%	86.97%	0.636	0.731	0.645	0.731
**Non-expert 1**	572	79.57%	85.78%	87.38%	88.79%	80.73%	89.11%	83.22%	88.99%	0.655	0.768	0.663	0.769
**Non-expert 2**	2,555	79.51%	83.19%	83.94%	84.83%	82.08%	87.40%	82.82%	86.38%	0.648	0.718	0.651	0.718
**Non-expert 3**	736	79.45%	82.99%	78.23%	82.99%	87.56%	88.69%	83.83%	86.41%	0.661	0.717	0.662	0.717
**Expert 21**	690	79.35%	82.57%	83.59%	85.88%	83.41%	86.45%	83.48%	86.23%	0.656	0.712	0.659	0.714
**Expert 22**	1,120	79.33%	84.42%	79.92%	84.78%	84.23%	88.25%	82.41%	86.79%	0.640	0.730	0.640	0.730
**Expert 23**	722	79.08%	82.37%	92.11%	84.59%	74.27%	86.91%	81.16%	86.01%	0.626	0.708	0.647	0.709
**Expert 24**	628	78.28%	82.47%	86.36%	85.54%	78.50%	86.27%	81.53%	85.99%	0.625	0.708	0.633	0.710
**Expert 25**	585	78.10%	85.29%	92.13%	86.89%	63.21%	85.85%	76.41%	86.32%	0.538	0.725	0.568	0.726
**Non-expert 4**	628	77.95%	85.18%	80.08%	88.67%	82.53%	86.56%	81.53%	87.42%	0.621	0.743	0.621	0.745
**Expert 26**	913	77.70%	85.06%	74.81%	86.38%	86.83%	87.60%	81.71%	87.08%	0.622	0.737	0.624	0.737
**Non-expert 5**	564	77.54%	81.93%	79.22%	80.00%	79.29%	87.38%	79.26%	84.04%	0.583	0.677	0.583	0.677
**Expert 27**	619	77.41%	79.58%	77.73%	79.41%	85.56%	87.40%	82.55%	84.33%	0.632	0.669	0.632	0.669
**Non-expert 6**	610	77.38%	82.62%	82.63%	85.59%	80.48%	86.36%	81.31%	86.07%	0.616	0.710	0.619	0.711
**Expert 28**	865	76.92%	83.17%	85.29%	86.47%	76.38%	86.10%	79.88%	86.24%	0.594	0.716	0.603	0.717
**Non-expert 7**	644	76.82%	84.39%	83.15%	85.02%	76.39%	88.33%	79.19%	86.96%	0.581	0.732	0.587	0.732
**Non-expert 8**	603	76.39%	83.27%	79.28%	85.26%	79.83%	86.08%	79.60%	85.74%	0.585	0.709	0.586	0.709
**Non-expert 9**	622	76.25%	82.00%	72.08%	83.40%	87.39%	85.15%	80.87%	84.41%	0.603	0.683	0.606	0.683
**Expert 29**	723	76.19%	82.67%	81.08%	83.78%	77.99%	86.89%	79.25%	85.62%	0.579	0.704	0.583	0.704
**Expert 30**	597	76.05%	81.67%	89.27%	84.12%	70.88%	85.99%	78.06%	85.26%	0.566	0.694	0.588	0.694
**Non-expert 10**	586	75.78%	82.23%	83.98%	83.12%	75.49%	87.61%	78.84%	85.84%	0.573	0.705	0.582	0.705
**Non-expert 11**	594	75.74%	84.03%	70.17%	84.03%	89.89%	89.33%	81.99%	87.21%	0.616	0.734	0.621	0.734
**Non-expert 12**	606	75.70%	83.04%	81.27%	84.86%	76.34%	86.20%	78.38%	85.64%	0.564	0.706	0.568	0.707
**Non-expert 13**	703	75.45%	82.54%	89.77%	83.50%	63.50%	85.75%	74.82%	84.78%	0.509	0.691	0.537	0.691
**Non-expert 14**	580	75.25%	85.12%	78.24%	86.19%	79.18%	88.56%	78.79%	87.59%	0.568	0.745	0.569	0.745
**Non-expert 15**	1,149	75.13%	83.30%	78.08%	85.91%	81.05%	87.04%	79.90%	86.60%	0.583	0.721	0.584	0.722
**Non-expert 16**	655	74.96%	83.68%	84.23%	86.38%	69.95%	85.11%	76.03%	85.65%	0.525	0.709	0.537	0.710
**Non-expert 17**	622	74.78%	85.50%	95.45%	87.12%	55.87%	87.71%	72.67%	87.46%	0.479	0.745	0.535	0.745
**Expert 31**	892	74.52%	82.67%	71.88%	86.08%	86.30%	85.56%	80.61%	85.76%	0.589	0.706	0.590	0.708
**Expert 32**	886	74.43%	82.27%	64.79%	83.66%	93.79%	86.82%	82.17%	85.55%	0.612	0.701	0.628	0.701
**Non-expert 18**	649	74.25%	80.89%	87.08%	83.75%	72.13%	86.31%	77.66%	85.36%	0.553	0.691	0.572	0.692
**Non-expert 19**	770	74.19%	81.93%	83.76%	83.76%	71.05%	85.75%	76.23%	84.94%	0.527	0.690	0.539	0.691
**Non-expert 20**	583	74.04%	84.73%	92.86%	87.39%	60.00%	86.96%	73.41%	87.14%	0.490	0.736	0.534	0.737
**Non-expert 21**	574	73.98%	82.15%	78.45%	82.33%	77.19%	87.72%	77.70%	85.54%	0.546	0.700	0.549	0.700
**Expert 33**	682	73.29%	84.08%	79.51%	85.87%	73.43%	86.97%	75.95%	86.51%	0.517	0.724	0.522	0.724
**Non-expert 22**	598	73.16%	86.58%	75.09%	86.42%	75.98%	89.49%	75.59%	88.13%	0.508	0.759	0.509	0.759
**Non-expert 23**	673	73.08%	83.42%	71.43%	83.28%	82.12%	87.82%	77.56%	85.88%	0.539	0.711	0.539	0.711
**Non-expert 24**	694	72.73%	82.17%	89.36%	83.33%	61.41%	86.65%	72.77%	85.30%	0.474	0.697	0.507	0.697
**Non-expert 25**	628	72.42%	84.40%	87.35%	86.12%	65.54%	88.51%	74.04%	87.58%	0.492	0.741	0.519	0.741
**Non-expert 26**	607	71.91%	82.22%	75.89%	84.98%	74.86%	84.46%	75.29%	84.68%	0.500	0.688	0.502	0.689
**Non-expert 27**	609	71.86%	82.47%	70.64%	85.11%	83.69%	86.63%	78.65%	86.04%	0.547	0.709	0.547	0.710
**Non-expert 28**	612	70.18%	82.44%	66.28%	82.76%	83.19%	86.61%	75.98%	84.97%	0.502	0.693	0.504	0.693
**Non-expert 29**	583	69.55%	80.93%	85.78%	84.89%	61.73%	84.36%	71.01%	84.56%	0.437	0.680	0.467	0.682
**Non-expert 30**	568	67.96%	84.68%	69.30%	87.28%	76.76%	87.35%	73.77%	87.32%	0.458	0.739	0.458	0.740
**Non-expert 31**	579	67.82%	84.43%	56.61%	85.12%	92.58%	88.13%	77.55%	86.87%	0.516	0.731	0.541	0.731
**Non-expert 32**	1,501	63.52%	84.43%	51.97%	85.20%	92.05%	88.69%	75.82%	87.28%	0.467	0.737	0.494	0.737
**Non-expert 33**	578	60.30%	83.29%	56.60%	83.49%	81.97%	90.16%	72.66%	87.72%	0.396	0.736	0.398	0.736

Kappa = Cohen's kappa coefficient. MCC = Matthew's correlation coefficient.

Supplementary Table 3 | Performance of AI models and human examiners by center

Centre	Cases		F1 score	Sensitivity	Specificity	Accuracy	Kappa	MCC	AUC	Brier score
**Ancona, Italy**	90 (51 benign, 39 malignant)	AI models	80.00% (68.35–89.16)	71.79% (56.76–85.37)	94.12% (86.79–100.00)	84.44% (76.67–91.11)	0.675 (0.509–0.817)	0.687 (0.530–0.824)	0.945 (0.891–0.986)	0.105 (0.075–0.139)
		Experts	73.53% (60.60–84.51)	64.10% (50.00–80.00)	92.16% (82.76–98.08)	90.00% (71.11–87.78)	0.580 (0.398–0.743)	0.597 (0.418–0.752)		
		Non-experts	66.67% (52.94–78.57)	61.54% (44.68–75.68)	84.31% (74.51–94.12)	74.44% (64.44–83.33)	0.465 (0.270–0.640)	0.475 (0.279–0.649)		
**Athens, Greece**	83 (47 benign, 36 malignant)	AI models	86.49% (76.92–93.98)	88.89% (77.50–97.44)	87.23% (76.92–95.83)	87.95% (80.72–93.98)	0.756 (0.604–0.880)	0.757 (0.608–0.885)	0.931 (0.862–0.981)	0.098 (0.064–0.138)
		Experts	86.11% (76.06–93.75)	83.33% (70.59–94.59)	91.49% (82.50–98.04)	87.95% (80.72–95.18)	0.755 (0.601–0.880)	0.755 (0.606–0.886)		
		Non-experts	81.08% (68.97–89.55)	80.56% (67.50–93.02)	85.11% (73.81–94.00)	83.13% (74.70–90.36)	0.659 (0.479–0.807)	0.660 (0.483–0.808)		
**Barcelona, Spain**	114 (58 benign, 56 malignant)	AI models	84.11% (75.79–90.91)	80.36% (69.81–90.16)	89.66% (81.03–96.67)	85.09% (78.07–91.23)	0.701 (0.561–0.824)	0.704 (0.567–0.825)	0.899 (0.837–0.951)	0.128 (0.098–0.160)
		Experts	79.63% (69.90–86.96)	78.57% (65.45–87.69)	82.76% (72.73–92.06)	80.70% (72.81–86.84)	0.613 (0.443–0.738)	0.614 (0.448–0.746)		
		Non-experts	74.29% (64.65–82.88)	73.21% (60.71–84.31)	77.59% (66.67–88.33)	75.44% (67.54–83.33)	0.508 (0.346–0.661)	0.509 (0.348–0.664)		
**Bologna, Italy**	179 (104 benign, 75 malignant)	AI models	73.53% (64.57–81.20)	66.67% (56.06–77.27)	89.42% (82.95–95.00)	79.89% (73.74–85.47)	0.576 (0.452–0.692)	0.584 (0.461–0.699)	0.855 (0.794–0.909)	0.151 (0.122–0.182)
		Experts	72.97% (64.79–80.77)	73.33% (62.86–82.90)	81.73% (73.33–88.29)	77.65% (71.51–83.80)	0.538 (0.416–0.662)	0.538 (0.417–0.664)		
		Non-experts	66.23% (56.72–74.53)	66.67% (55.55–77.14)	75.00% (66.39–83.18)	72.07% (64.80–78.21)	0.426 (0.276–0.547)	0.426 (0.276–0.548)		
**Brescia, Italy**	161 (131 benign, 30 malignant)	AI models	77.19% (63.16–88.14)	73.33% (56.52–88.89)	96.18% (92.59–99.22)	91.93% (87.58–95.65)	0.723 (0.561–0.854)	0.725 (0.566–0.855)	0.921 (0.853–0.970)	0.086 (0.063–0.112)
		Experts	64.41% (50.79–78.69)	63.33% (47.37–81.48)	93.13% (87.90–96.95)	86.96% (81.99–92.55)	0.568 (0.408–0.737)	0.570 (0.412–0.738)		
		Non-experts	55.38% (40.00–69.23)	60.00% (42.31–78.57)	87.02% (80.83–92.31)	81.99% (75.78–88.20)	0.442 (0.266–0.610)	0.444 (0.268–0.616)		
**Cagliari, Italy**	90 (75 benign, 15 malignant)	AI models	75.00% (54.55–90.00)	80.00% (57.14–100.00)	93.33% (87.18–98.63)	91.11% (84.44–96.67)	0.686 (0.468–0.877)	0.698 (0.472–0.879)	.922 (0.767– 0.997)	0.074 (0.050–0.104)
		Experts	75.86% (55.56–90.00)	80.00% (60.00–100.00)	92.00% (86.25–97.47)	91.11% (84.44–96.67)	0.711 (0.475–0.877)	0.713 (0.492–0.877)		
		Non-experts	62.50% (41.38–80.00)	73.33% (50.00–94.12)	88.00% (80.26–94.67)	85.56% (77.78–92.22)	0.541 (0.308–0.752)	0.546 (0.322–0.758)		
**Katowice, Poland**	111 (55 benign, 56 malignant)	AI models	95.58% (91.09–99.08)	96.43% (90.74–100.00)	94.55% (87.72–100.00)	95.50% (90.99–99.10)	0.910 (0.820–0.982)	0.910 (0.822–0.982)	0.977 (0.947–0.997)	0.067 (0.047–0.091)
		Experts	91.07% (84.96–96.00)	91.07% (82.35–97.96)	92.73% (83.64–98.21)	90.99% (85.59–96.40)	0.820 (0.710–0.925)	0.820 (0.711–0.925)		
		Non-experts	89.09% (81.63–94.21)	89.29% (79.99–96.36)	89.09% (78.95–96.08)	89.19% (81.98–93.69)	0.784 (0.639–0.874)	0.784 (0.640–0.875)		
**Kaunas, Lithuania**	132 (66 benign, 66 malignant)	AI models	86.61% (79.66–92.31)	83.33% (73.85–91.89)	90.91% (83.33–97.06)	87.12% (81.06–92.42)	0.742 (0.621–0.848)	0.745 (0.624–0.849)	0.952 (0.915–0.981)	0.098 (0.075–0.124)
		Experts	83.33% (75.97–89.92)	81.82% (72.88–91.53)	84.85% (75.38–92.75)	83.33% (77.27–90.15)	0.667 (0.543–0.797)	0.668 (0.543–0.798)		
		Non-experts	78.12% (69.35–84.89)	78.79% (68.63–88.24)	77.27% (65.57–86.11)	78.03% (70.45–84.09)	0.561 (0.405–0.682)	0.561 (0.406–0.685)		
**Lublin, Poland**	211 (124 benign, 87 malignant)	AI models	80.87% (74.12–86.86)	85.06% (77.01–92.22)	82.26% (75.57–88.79)	83.41% (78.20–88.63)	0.663 (0.558–0.762)	0.665 (0.562–0.765)	0.897 (0.852–0.937)	0.130 (0.103–0.158)
		Experts	73.80% (66.67–80.85)	79.31% (70.59–87.78)	74.19% (67.46–82.71)	76.78% (71.09–82.46)	0.534 (0.420–0.649)	0.538 (0.427–0.652)		
		Non-experts	69.84% (61.80–76.70)	75.86% (66.67–85.06)	70.16% (62.07–78.05)	72.51% (66.35–78.67)	0.452 (0.329–0.566)	0.460 (0.335–0.573)		
**Lund, Sweden**	100 (51 benign, 49 malignant)	AI models	85.15% (76.92–91.84)	87.76% (78.00–96.00)	82.35% (70.83–92.00)	85.00% (78.00–92.00)	0.700 (0.552–0.835)	0.702 (0.556–0.835)	0.895 (0.827–0.951)	0.136 (0.106–0.169)
		Experts	79.61% (70.10–87.27)	83.67% (73.47–93.88)	72.55% (60.38–84.62)	79.00% (70.00–86.00)	0.581 (0.408–0.721)	0.587 (0.417–0.731)		
		Non-experts	75.73% (66.67–84.91)	79.59% (69.39–91.30)	70.59% (57.41–82.76)	75.00% (67.00–84.00)	0.501 (0.340–0.678)	0.503 (0.343–0.680)		
**Manila, Philippines**	97 (49 benign, 48 malignant)	AI models	83.81% (75.51–90.91)	91.67% (82.98–98.08)	73.47% (60.87–85.71)	82.47% (75.26–89.69)	0.650 (0.497–0.794)	0.662 (0.514–0.800)	0.944 (0.896–0.980)	0.110 (0.078–0.145)
		Experts	81.63% (72.50–89.11)	83.33% (72.55–93.48)	79.59% (66.67–89.80)	81.44% (73.20–88.66)	0.629 (0.464–0.773)	0.630 (0.465–0.774)		
		Non-experts	77.42% (65.78–84.68)	79.17% (65.38–88.68)	75.51% (61.11–85.71)	77.32% (67.01–83.51)	0.547 (0.340–0.672)	0.548 (0.342–0.680)		
**Milan, Italy**	118 (50 benign, 68 malignant)	AI models	89.66% (84.00–94.34)	95.59% (90.14–100.00)	76.00% (63.64–87.27)	87.29% (81.36–93.22)	0.733 (0.599–0.851)	0.743 (0.619–0.855)	0.935 (0.883–0.976)	0.101 (0.074–0.131)
		Experts	84.77% (77.86–90.32)	91.18% (83.58–97.10)	66.00% (53.85–80.00)	81.36% (73.73–87.29)	0.601 (0.446–0.737)	0.616 (0.459–0.745)		
		Non-experts	80.56% (72.87–87.01)	85.29% (76.56–93.42)	64.00% (50.00–77.08)	76.27% (68.64–83.90)	0.504 (0.339–0.656)	0.509 (0.347–0.662)		
**Milan 2, Italy**	350 (266 benign, 84 malignant)	AI models	78.31% (71.51–84.42)	88.10% (80.68–94.57)	88.35% (84.29–92.11)	88.29% (84.86–91.43)	0.704 (0.618–0.785)	0.712 (0.629–0.790)	0.944 (0.919–0.966)	0.098 (0.081–0.116)
		Experts	71.20% (64.00–78.39)	80.95% (71.25–88.46)	86.09% (81.89–90.16)	84.57% (80.86–88.29)	0.606 (0.518–0.702)	0.615 (0.526–0.708)		
		Non-experts	62.69% (54.78–70.29)	75.00% (64.63–83.56)	80.45% (75.48–84.94)	78.86% (74.57–83.14)	0.482 (0.386–0.583)	0.495 (0.397–0.593)		
**Monza, Italy**	161 (102 benign, 59 malignant)	AI models	78.50% (68.89–86.49)	71.19% (59.26–82.76)	94.12% (89.11–98.10)	85.71% (80.12–90.68)	0.680 (0.550–0.795)	0.688 (0.563–0.801)	0.960 (0.931–0.983)	0.095 (0.075–0.118)
		Experts	83.48% (75.81–90.23)	83.05% (73.53–92.31)	91.18% (84.76–96.08)	88.20% (82.61–92.55)	0.741 (0.628–0.844)	0.743 (0.629–0.845)		
		Non-experts	77.59% (68.04–85.11)	76.27% (65.52–87.10)	87.25% (80.65–93.48)	83.85% (77.64–88.82)	0.650 (0.513–0.761)	0.652 (0.515–0.762)		
**Pamplona, Spain**	77 (47 benign, 30 malignant)	AI models	89.66% (80.00–96.77)	86.67% (73.33–96.97)	95.74% (89.19–100.00)	92.21% (85.71–97.40)	0.834 (0.695–0.946)	0.835 (0.699–0.947)	0.962 (0.910–0.996)	0.072 (0.043–0.106)
		Experts	82.76% (69.38–91.80)	76.67% (61.54–91.67)	93.62% (84.44–100.00)	87.01% (79.22–93.51)	0.724 (0.541–0.866)	0.725 (0.548–0.869)		
		Non-experts	75.47% (61.54–86.96)	70.00% (54.29–87.10)	89.36% (79.55–97.62)	81.82% (74.03–90.91)	0.618 (0.423–0.787)	0.620 (0.430–0.791)		
**Prague, Czech Republic**	105 (35 benign, 70 malignant)	AI models	88.24% (81.97–93.43)	85.71% (76.92–93.42)	82.86% (69.23–94.29)	84.76% (77.14–91.43)	0.667 (0.511–0.806)	0.669 (0.517–0.809)	0.946 (0.898–0.982)	0.103 (0.079–0.131)
		Experts	84.56% (77.27–90.12)	88.57% (79.69–95.08)	57.14% (41.67–75.00)	78.10% (69.52–85.71)	0.481 (0.288–0.658)	0.487 (0.296–0.664)		
		Non-experts	82.12% (74.29–88.05)	87.14% (77.94–94.29)	51.43% (33.33–66.67)	74.29% (65.71–82.86)	0.391 (0.190–0.566)	0.396 (0.195–0.577)		
**Prague 2, Czech Republic**	110 (64 benign, 46 malignant)	AI models	82.11% (72.92–89.80)	84.78% (73.33–94.74)	84.38% (75.00–92.65)	84.55% (77.27–90.91)	0.685 (0.542–0.816)	0.686 (0.545–0.818)	0.921 (0.864–0.967)	0.118 (0.090–0.150)
		Experts	75.00% (64.44–84.21)	78.26% (66.00–90.00)	78.12% (67.19–87.69)	78.18% (70.00–85.45)	0.557 (0.397–0.709)	0.559 (0.400–0.713)		
		Non-experts	71.29% (59.70–80.67)	76.09% (63.04–88.10)	71.88% (60.87–82.81)	73.64% (65.45–81.82)	0.473 (0.307–0.636)	0.479 (0.311–0.638)		
**Stockholm, Sweden**	300 (150 benign, 150 malignant)	AI models	84.83% (80.40–88.77)	91.33% (86.52–95.54)	76.00% (68.87–82.67)	83.67% (79.33–87.67)	0.673 (0.587–0.753)	0.681 (0.597–0.759)	0.921 (0.889–0.948)	0.121 (0.102–0.142)
		Experts	81.66% (76.57–85.80)	89.33% (84.21–94.16)	69.33% (62.18–76.81)	79.67% (75.00–84.00)	0.593 (0.499–0.679)	0.608 (0.513–0.689)		
		Non-experts	79.04% (73.82–83.47)	89.33% (83.67–93.71)	63.33% (55.40–70.70)	76.33% (71.33–80.67)	0.527 (0.428–0.612)	0.545 (0.448–0.627)		
**Trieste, Italy**	71 (50 benign, 21 malignant)	AI models	79.17% (64.28–90.48)	90.48% (76.00–100.00)	84.00% (72.92–93.75)	85.92% (77.46–92.96)	0.688 (0.498–0.852)	0.700 (0.519–0.857)	0.950 (0.895–0.989)	0.099 (0.066–0.137)
		Experts	73.91% (57.89–86.96)	85.71% (69.22–100.00)	82.00% (70.00–91.67)	83.10% (73.24–91.55)	0.616 (0.412–0.795)	0.621 (0.433–0.804)		
		Non-experts	64.00% (46.15–77.42)	80.95% (60.00–95.00)	70.00% (58.00–83.02)	73.24% (63.38–83.10)	0.433 (0.226–0.639)	0.466 (0.247–0.656)		

Data in parentheses are 95% CIs through bootstrapping. Kappa = Cohen's kappa coefficient. MCC = Matthew's correlation coefficient.

Supplementary Table 4 | Performance of AI models and human examiners by ultrasound system

Ultrasound System	Cases		F1 score	Sensitivity	Specificity	Accuracy	Kappa	MCC	AUC	Brier score
**GE Voluson E6**	325 (200 benign, 125 malignant)	AI models	79.15% (73.15–84.43)	74.40% (66.42–81.91)	91.50% (87.44–95.15)	84.92% (80.92–88.62)	0.674 (0.588–0.752)	0.678 (0.593–0.756)	0.911 (0.875–0.943)	0.114 (0.096–0.134)
		Experts	80.00% (74.29–85.14)	79.20% (72.00–86.32)	88.00% (83.41–92.31)	84.62% (80.62–88.31)	0.676 (0.590–0.755)	0.676 (0.591–0.755)		
		Non-experts	73.75% (66.95–79.53)	73.60% (65.08–80.74)	84.00% (78.72–88.78)	80.00% (75.38–84.00)	0.578 (0.477–0.662)	0.579 (0.477–0.663)		
**GE Voluson E8**	1,074 (685 benign, 389 malignant)	AI models	82.81% (79.89–85.43)	84.83% (81.28–88.29)	88.61% (86.21–90.91)	87.24% (85.20–89.20)	0.727 (0.684–0.767)	0.727 (0.684–0.768)	0.925 (0.908–0.941)	0.106 (0.096–0.117)
		Experts	77.09% (73.67–79.95)	79.69% (75.41–83.38)	84.53% (81.92–87.28)	82.87% (80.45–84.92)	0.633 (0.583–0.677)	0.635 (0.585–0.678)		
		Non-experts	71.20% (67.41–74.51)	75.32% (70.57–79.34)	79.56% (76.49–82.56)	77.93% (75.42–80.35)	0.534 (0.480–0.584)	0.536 (0.483–0.586)		
**GE Voluson E10**	579 (287 benign, 292 malignant)	AI models	85.57% (82.51–88.40)	89.38% (85.71–92.86)	80.14% (75.34–84.56)	84.80% (81.87–87.56)	0.696 (0.636–0.751)	0.699 (0.640–0.754)	0.926 (0.905– 0.945)	0.116 (0.103–0.129)
		Experts	81.59% (77.99–84.56)	88.01% (84.05–91.55)	71.78% (66.10–76.67)	79.97% (76.51–82.90)	0.599 (0.529–0.658)	0.606 (0.537–0.664)		
		Non-experts	78.07% (74.50–81.54)	86.30% (82.27–90.12)	64.81% (59.18–70.29)	75.65% (72.19–79.10)	0.512 (0.443–0.578)	0.523 (0.455–0.590)		
**GE Voluson I**	89 (74 benign, 15 malignant)	AI models	75.00% (54.55–89.47)	80.00% (57.14–100.00)	93.24% (86.96–98.63)	91.01% (84.27–96.63)	0.695 (0.468–0.869)	0.697 (0.482–0.870)	0.922 (0.770–0.997)	0.074 (0.050–0.104)
		Experts	75.86% (54.55–89.66)	80.00% (60.00–100.00)	91.89% (86.08–97.50)	91.01% (84.27–96.63)	0.710 (0.468–0.871)	0.712 (0.482–0.875)		
		Non-experts	62.50% (42.09–80.00)	73.33% (50.00–94.44)	87.84% (80.28–94.67)	85.39% (78.65–92.13)	0.540 (0.313–0.751)	0.545 (0.325–0.758)		
**GE Voluson S10**	124 (72 benign, 52 malignant)	AI models	83.48% (75.27–90.27)	92.31% (84.31–98.28)	79.17% (69.23–88.16)	84.68% (78.23–91.13)	0.694 (0.563–0.817)	0.705 (0.581–0.821)	0.954 (0.915–0.983)	0.105 (0.078–0.134)
		Experts	79.28% (70.37–87.18)	82.69% (72.34–92.45)	80.56% (71.83–89.71)	81.45% (75.00–88.71)	0.626 (0.494–0.767)	0.630 (0.497–0.769)		
		Non-experts	73.87% (62.92–81.55)	76.92% (65.52–88.46)	76.39% (64.79–84.72)	77.42% (68.55–83.06)	0.536 (0.359–0.661)	0.536 (0.364–0.666)		
**GE Voluson 730**	169 (89 benign, 90 malignant)	AI models	88.31% (82.35–93.33)	85.00% (76.62–92.41)	93.26% (87.63–97.89)	89.35% (84.62–94.08)	0.786 (0.687–0.876)	0.788 (0.691–0.877)	0.956 (0.926–0.981)	0.093 (0.073–0.115)
		Experts	85.71% (78.83–90.70)	83.75% (75.31–91.67)	88.76% (81.25–94.74)	86.98% (81.07–91.12)	0.738 (0.611–0.822)	0.739 (0.615–0.823)		
		Non-experts	70.00% (72.05–86.02)	81.25% (71.21–88.64)	80.90% (71.76–88.37)	80.07% (73.96–86.39)	0.619 (0.480–0.727)	0.620 (0.482–0.727)		
**Mindray DC-70**	44 (17 benign, 27 malignant)	AI models	72.73% (55.81–86.21)	59.26% (40.62–77.78)	94.12% (80.00–100.00)	72.73% (59.09–86.36)	0.481 (0.255–0.715)	0.534 (0.309–0.728)	0.919 (0.826–0.987)	0.148 (0.094–0.210)
		Experts	74.42% (57.89–87.27)	59.26% (41.67–78.57)	100.00% (86.67–100.00)	75.00% (61.36–86.36)	0.520 (0.298–0.732)	0.571 (0.375–0.760)		
		Non-experts	69.57% (50.00–82.61)	55.56% (35.71–73.91)	88.24% (73.33–100.00)	68.18% (54.55–81.82)	0.395 (0.169–0.642)	0.442 (0.208–0.667)		
**Samsung WS80A**	159 (104 benign, 55 malignant)	AI models	86.67% (79.28–92.68)	94.55% (87.72–100.00)	87.50% (80.73–93.52)	89.94% (84.91–94.34)	0.787 (0.681–0.881)	0.794 (0.694–0.885)	0.958 (0.926–0.983)	0.089 (0.067–0.114)
		Experts	79.67% (70.67–86.36)	89.09% (80.33–96.43)	81.73% (73.12–88.24)	84.28% (77.99–89.31)	0.671 (0.541–0.773)	0.681 (0.556–0.780)		
		Non-experts	73.77% (64.29–81.63)	87.27% (76.92–94.83)	74.04% (66.00–82.52)	79.25% (71.70–84.91)	0.568 (0.432–0.685)	0.584 (0.454–0.698)		

Data in parentheses are 95% CIs through bootstrapping. The table is limited to the eight most common ultrasound systems in the OMLC-RS dataset. Kappa = Cohen's kappa coefficient. MCC = Matthew's correlation coefficient.

Supplementary Table 5 | Performance of AI models and human examiners by level of confidence in examiners' assessments

Confidence		F1 score	Sensitivity	Specificity	Accuracy	Kappa	MCC	AUC	Brier score
**Certain** ***n*** **= 10,079**	AI models	87.08% (86.28–87.88)	88.98% (87.96–89.94)	90.77% (90.04–91.46)	90.10% (89.50–90.68)	0.791 (0.778–0.803)	0.791 (0.779–0.803)	0.952 (0.948–0.957)	0.084 (0.081–0.087)
	Non-expert	84.24% (83.36–85.09)	87.26% (86.17–88.31)	88.04% (87.23–88.85)	87.75% (87.10–88.38)	0.742 (0.729–0.756)	0.744 (0.730–0.757)		
**Probable** ***n*** **= 10,951**	AI models	81.19% (80.35–82.03)	82.44% (81.36–83.51)	84.80% (83.93–85.68)	83.80% (83.12–84.49)	0.670 (0.656–0.684)	0.670 (0.656–0.684)	0.909 (0.904–0.914)	0.124 (0.120–0.127)
	Non-experts	70.14% (69.11–71.15)	75.25% (74.01–76.50)	71.04% (69.92–72.16)	72.82% (71.98–73.66)	0.454 (0.438–0.471)	0.458 (0.441–0.474)		
**Uncertain** ***n*** **= 2,805**	AI models	80.63% (78.95–82.27)	81.66% (79.52–83.76)	82.96% (81.07–84.79)	82.38% (80.93–83.74)	0.645 (0.616–0.672)	0.645 (0.616–0.673)	0.899 (0.887–0.910)	0.131 (0.125–0.137)
	Non-experts	59.83% (57.63–61.97)	64.62% (61.90–67.28)	58.09% (55.58–60.61)	61.03% (59.18–62.85)	0.224 (0.187–0.260)	0.226 (0.190–0.262)		
**Certain** ***n*** **= 16,209**	AI models	88.69% (88.11–89.25)	90.25% (89.50–90.96)	91.42% (90.87–91.98)	90.96% (90.52–91.40)	0.812 (0.803–0.821)	0.812 (0.803–0.821)	0.957 (0.954–0.960)	0.079 (0.077–0.082)
	Experts	88.21% (87.62–88.78)	90.58% (89.87–91.29)	90.43% (89.85–91.00)	90.49% (90.04–90.94)	0.802 (0.793–0.812)	0.803 (0.794–0.812)		
**Probable** ***n*** **= 9,516**	AI models	77.83% (76.83–78.82)	79.20% (77.94–80.45)	82.31% (81.27–83.32)	81.00% (80.21–81.79)	0.612 (0.596–0.628)	0.612 (0.596–0.629)	0.880 (0.873–0.887)	0.142 (0.138–0.145)
	Experts	70.40% (69.29–71.49)	74.15% (72.80–75.50)	73.43% (72.27–74.58)	73.73% (72.86–74.60)	0.469 (0.451–0.486)	0.471 (0.453–0.489)		
**Uncertain** ***n*** **= 1,619**	AI models	75.33% (72.87–77.70)	76.05% (72.89–79.04)	77.99% (75.20–80.68)	77.10% (75.05–79.06)	0.539 (0.498–0.579)	0.540 (0.498–0.579)	0.844 (0.825–0.862)	0.162 (0.153–0.171)
	Experts	59.77% (56.85–62.61)	61.46% (57.99–64.93)	62.38% (59.19–65.58)	61.96% (59.67–64.30)	0.237 (0.191–0.285)	0.238 (0.191–0.285)		

Data in parentheses are 95% CIs through bootstrapping. Kappa = Cohen's kappa coefficient. MCC = Matthew's correlation coefficient.

Supplementary Table 6 | Performance of AI models and human examiners by patient age group

Age group	Cases		F1 score	Sensitivity	Specificity	Accuracy	Kappa	MCC	AUC	Brier score
**≤ 29**	315 (243 benign, 72 malignant)	AI models	67.10% (57.97–75.34)	72.22% (61.43–82.35)	87.24% (82.92–91.29)	83.81% (79.68–87.94)	0.564 (0.454–0.669)	0.567 (0.458–0.672)	0.888 (0.845–0.927)	0.119 (0.100–0.139)
		Experts	65.41% (55.70–72.63)	75.00% (64.06–84.21)	83.54% (78.51–87.97)	81.59% (77.14–85.71)	0.531 (0.416–0.626)	0.543 (0.426–0.632)		
		Non-experts	57.29% (47.90–65.33)	72.22% (62.16–82.86)	76.13% (70.20–81.05)	75.24% (70.16–79.68)	0.408 (0.299–0.510)	0.428 (0.318–0.529)		
**30–39**	447 (348 benign, 99 malignant)	AI models	78.14% (71.56–83.87)	84.85% (77.45–91.67)	90.80% (87.64–93.71)	89.49% (86.58–92.17)	0.713 (0.632–0.786)	0.717 (0.638–0.789)	0.939 (0.911–0.963)	0.091 (0.078–0.106)
		Experts	71.80% (64.11–77.39)	81.82% (75.23–90.00)	85.63% (81.98–89.36)	85.01% (81.66–88.37)	0.610 (0.529–0.693)	0.620 (0.543–0.702)		
		Non-experts	60.41% (52.94–67.19)	75.76% (67.39–84.16)	78.16% (73.95–82.70)	77.85% (74.05–81.66)	0.460 (0.372–0.546)	0.480 (0.391–0.565)		
**40–49**	526 (317 benign, 209 malignant)	AI models	84.04% (80.09–87.58)	85.65% (80.75–90.32)	88.01% (84.30–91.44)	87.07% (84.22–89.92)	0.732 (0.670–0.789)	0.732 (0.671–0.790)	0.924 (0.899–0.945)	0.110 (0.096–0.125)
		Experts	80.47% (76.12–84.21)	83.25% (77.78–88.12)	84.23% (80.06–88.13)	83.84% (80.61–86.88)	0.668 (0.599–0.729)	0.670 (0.601–0.730)		
		Non-experts	75.06% (70.67–79.57)	79.90% (74.02–84.89)	78.86% (74.27–83.33)	79.09% (75.67–82.51)	0.572 (0.502–0.643)	0.574 (0.505–0.645)		
**50–59**	470 (266 benign, 204 malignant)	AI models	85.51% (81.82–88.99)	88.24% (83.70–92.49)	86.09% (81.89–90.16)	87.02% (84.04–90.00)	0.738 (0.675–0.799)	0.739 (0.678–0.799)	0.931 (0.907–0.954)	0.107 (0.093–0.122)
		Experts	79.34% (74.76–83.33)	82.35% (76.92–87.50)	80.45% (75.66–85.09)	81.28% (77.66–84.68)	0.624 (0.550–0.692)	0.626 (0.552–0.693)		
		Non-experts	73.66% (69.06–78.24)	78.92% (72.77–83.92)	74.06% (68.54–79.25)	75.53% (71.91–79.79)	0.512 (0.438–0.592)	0.516 (0.442–0.595)		
**60–69**	401 (180 benign, 221 malignant)	AI models	84.45% (81.09–88.31)	82.35% (77.29–87.14)	85.56% (80.23–90.52)	83.79% (80.05–87.28)	0.675 (0.600–0.745)	0.676 (0.603–0.746)	0.908 (0.878–0.935)	0.122 (0.104–0.141)
		Experts	80.82% (76.61–84.62)	80.09% (74.65–85.17)	77.78% (71.68–83.89)	79.05% (75.06–83.04)	0.577 (0.496–0.656)	0.577 (0.497–0.657)		
		Non-experts	77.88% (73.43–82.02)	77.38% (71.37–82.41)	74.44% (68.10–80.85)	75.81% (71.82–80.05)	0.510 (0.430–0.599)	0.510 (0.430–0.599)		
**70 ≤**	292 (113 benign, 179 malignant)	AI models	88.51% (84.78–91.88)	86.03% (80.85–90.96)	86.73% (80.19–92.62)	86.30% (82.19–90.07)	0.716 (0.630–0.795)	0.718 (0.633–0.797)	0.947 (0.921–0.970)	0.098 (0.081–0.115)
		Experts	86.59% (82.70–90.16)	86.03% (80.81–90.91)	79.65% (72.12–87.04)	83.56% (79.45–87.67)	0.657 (0.558–0.735)	0.658 (0.559–0.736)		
		Non-experts	84.21% (80.12–88.39)	81.56% (75.84–87.08)	82.30% (74.07–88.39)	81.16% (77.05–85.96)	0.612 (0.518–0.702)	0.615 (0.522–0.704)		

Data in parentheses are 95% CIs through bootstrapping. Kappa = Cohen's kappa coefficient. MCC = Matthew's correlation coefficient. Information on patient age was missing for 125 patients.

Supplementary Table 7 | Performance of AI models and human examiners by year of examination

Year of examination	Cases		F1 score	Sensitivity	Specificity	Accuracy	Kappa	MCC	AUC	Brier score
**2006–2014**	493 (301 benign, 192 malignant)	AI models	84.24% (80.10–87.94)	84.90% (79.69–89.78)	89.37% (85.71–92.71)	87.63% (84.58–90.47)	0.741 (0.677–0.799)	0.741 (0.677–0.799)	0.935 (0.910–0.956)	0.101 (0.088–0.116)
		Experts	80.00% (75.54–84.24)	80.21% (75.00–86.14)	86.71% (82.71–90.37)	84.38% (81.14–87.63)	0.672 (0.604–0.738)	0.672 (0.604–0.738)		
		Non-experts	74.75% (69.85–79.41)	77.60% (71.43–83.33)	81.06% (76.56–85.47)	79.51% (76.06–83.16)	0.577 (0.503–0.650)	0.578 (0.505–0.652)		
**2015–2017**	556 (349 benign, 207 malignant)	AI models	82.78% (78.70–86.55)	83.57% (78.39–88.63)	89.11% (85.75–92.33)	87.05% (84.17–89.75)	0.724 (0.663–0.782)	0.724 (0.664–0.782)	0.919 (0.894–0.942)	0.109 (0.094–0.124)
		Experts	77.52% (72.86–81.58)	80.19% (74.34–85.34)	83.95% (80.24–87.89)	82.55% (79.50–85.61)	0.634 (0.566–0.696)	0.635 (0.567–0.697)		
		Non-experts	70.37% (65.43–75.11)	73.91% (67.89–79.80)	78.51% (74.25–82.91)	76.80% (73.38–80.40)	0.515 (0.442–0.586)	0.517 (0.444–0.588)		
**2018**	405 (233 benign, 172 malignant)	AI models	82.87% (78.49–86.84)	87.21% (81.98–92.03)	82.83% (77.83–87.50)	84.69% (81.23–88.15)	0.691 (0.619–0.758)	0.694 (0.624–0.761)	0.919 (0.890–0.943)	0.118 (0.101–0.136)
		Experts	80.22% (75.74–84.47)	86.05% (80.33–90.80)	79.40% (74.37–84.58)	81.98% (78.52–85.68)	0.639 (0.568–0.712)	0.644 (0.573–0.716)		
		Non-experts	74.48% (69.54–79.43)	81.98% (75.90–87.50)	72.96% (66.53–78.17)	76.05% (72.34–80.49)	0.524 (0.446–0.611)	0.533 (0.455–0.617)		
**2019**	478 (260 benign, 218 malignant)	AI models	84.06% (80.20–87.68)	83.49% (78.38–88.29)	87.31% (83.21–91.24)	85.56% (82.43–88.70)	0.709 (0.643–0.771)	0.709 (0.644–0.772)	0.927 (0.902–0.948)	0.109 (0.094–0.125)
		Experts	79.91% (76.04–84.10)	80.28% (75.34–85.71)	82.69% (78.26–87.45)	81.59% (78.45–85.15)	0.629 (0.564–0.702)	0.629 (0.565–0.703)		
		Non-experts	75.85% (71.39–80.35)	77.06% (71.36–82.67)	78.85% (73.31–83.27)	77.82% (74.06–81.59)	0.553 (0.477–0.628)	0.553 (0.478–0.628)		
**2020–2021**	728 (432 benign, 296 malignant)	AI models	83.50% (80.20–86.62)	85.47% (81.29–89.38)	86.81% (83.56–89.93)	86.26% (83.79–88.74)	0.717 (0.666–0.768)	0.718 (0.667–0.769)	0.939 (0.923–0.955)	0.104 (0.094–0.116)
		Experts	79.17% (75.66–82.64)	84.12% (79.81–88.22)	80.79% (76.84–84.31)	82.01% (79.26–84.89)	0.634 (0.579–0.692)	0.637 (0.583–0.695)		
		Non-experts	74.33% (70.48–78.01)	80.74% (76.37–85.46)	74.31% (70.52–78.77)	77.20% (74.18–80.22)	0.541 (0.480–0.602)	0.548 (0.487–0.607)		

Data in parentheses are 95% CIs through bootstrapping. Kappa = Cohen's kappa coefficient. MCC = Matthew's correlation coefficient.

Supplementary Table 8 | Performance of AI model on a separate cohort of cases from the Stockholm center

	**Remaining cases (*n* = 644)**	**Main analysis (*n* = 300)**
**F1 score**	84.73% (81.59–87.64)	84.83% (80.40–88.77)
**Sensitivity**	92.81% (89.66–95.67)	91.33% (86.52–95.54)
**Specificity**	80.05% (75.85–84.06)	76.00% (68.87–82.67)
**Accuracy**	85.56% (82.76–88.20)	83.67% (79.33–87.67)
**AUC**	0.954 (0.939–0.968)	0.921 (0.889–0.948)
**Kappa**	0.712 (0.658–0.765)	0.673 (0.587–0.753)
**MCC**	0.722 (0.671–0.772)	0.681 (0.597–0.759)
**Brier score**	0.102 (0.090–0.115)	0.121 (0.102–0.142)
**DOR**	51.78 (32.51–94.76)	33.37 (17.91–75.02)
**J**	72.86% (67.71–77.84)	67.33% (58.84–75.23)
**LR+**	4.65 (3.84–5.84)	3.81 (2.92–5.28)
**LR-**	0.090 (0.054–0.129)	0.114 (0.059–0.180)
**PPV**	77.95% (73.46–82.32)	79.19% (73.02–85.06)
**NPV**	93.61% (90.71–96.17)	89.76% (84.14–94.66)

Performance of the AI model on the cohort of 644 patients with a post-surgical histological diagnosis (366 benign, 278 malignant) from the Stockholm center that were not included in the main analysis as they were not assessed by human examiners. Data in parentheses are 95% CIs through bootstrapping. Kappa = Cohen's kappa coefficient. MCC = Matthew's correlation coefficient. DOR = diagnostic odds ratio. J = Youden's J statistic. LR+ = Positive likelihood ratio. LR– = Negative likelihood ratio. PPV = Positive predictive value. NPV = Negative predictive value.

Supplementary Table 9 | Impact of label granularity on AI model performance

	2 classes	10 classes	18 classes
**F1 score**	82.22% (80.39–83.91)	83.50% (81.76–85.14)	82.70% (80.93–84.36)
**Sensitivity**	81.38% (79.00–83.58)	84.88% (82.73–86.96)	83.50% (81.26–85.65)
**Specificity**	88.57% (86.99–90.13)	87.30% (85.66–88.94)	87.30% (85.63–88.92)
**Accuracy**	85.64% (84.29–86.95)	86.32% (85.00–87.59)	85.75% (84.44–87.07)
**AUC**	0.928 (0.918–0.937)	0.929 (0.919–0.939)	0.930 (0.920–0.939)
**Kappa**	0.702 (0.674–0.729)	0.718 (0.691–0.745)	0.706 (0.678–0.733)
**MCC**	0.702 (0.674–0.729)	0.718 (0.691–0.745)	0.706 (0.679–0.733)
**Brier score**	0.137 (0.133–0.142)	0.108 (0.101–0.114)	0.106 (0.100–0.113)
**DOR**	33.88 (27.46–42.37)	38.61 (31.16–48.74)	34.80 (28.23–43.73)
**J**	69.95% (67.07–72.66)	72.19% (69.46–74.86)	70.80% (68.04–73.52)
**LR+**	7.12 (6.22–8.27)	6.68 (5.90–7.69)	6.58 (5.80–7.56)
**LR-**	0.210 (0.185–0.237)	0.173 (0.149–0.198)	0.189 (0.164–0.215)
**PPV**	83.07% (80.83–85.26)	82.16% (79.93–84.38)	81.92% (79.63–84.18)
**NPV**	87.35% (85.68–88.92)	89.34% (87.77–90.84)	88.478% (86.85–90.04)

AI models were trained using two, ten, or 18 classes. At test-time, the model makes a binary prediction by averaging all the benign and malignant scores, as described in the main paper. Data in parentheses are 95% CIs through bootstrapping. Kappa = Cohen's kappa coefficient. MCC = Matthew's correlation coefficient. DOR = diagnostic odds ratio. J = Youden's J statistic. LR+ = Positive likelihood ratio. LR– = Negative likelihood ratio. PPV = Positive predictive value. NPV = Negative predictive value.

Supplementary Table 10 | Impact of image cropping on AI model performance

	Cropped and artifacts removed	Cropped only	Auto-cropped with YOLO[2]	Uncropped
**F1 score**	83.50% (81.76–85.14)	82.88% (81.12–84.52)	82.78% (81.04–84.41)	81.85% (80.09–83.53)
**Sensitivity**	84.88% (82.73–86.96)	85.44% (83.29–87.46)	85.71% (83.58–87.73)	84.61% (82.46–86.69)
**Specificity**	87.30% (85.66–88.94)	85.71% (84.02–87.44)	85.27% (83.55–87.02)	84.76% (82.98–86.52)
**Accuracy**	86.32% (85.00–87.59)	85.60% (84.25–86.92)	85.45% (84.10–86.80)	84.70% (83.31–86.05)
**AUC**	0.929 (0.919–0.939)	0.926 (0.915–0.936)	0.926 (0.916–0.936)	0.918 (0.907–0.928)
**Kappa**	0.718 (0.691–0.745)	0.705 (0.677–0.731)	0.702 (0.674–0.729)	0.687 (0.658–0.714)
**MCC**	0.718 (0.691–0.745)	0.706 (0.678–0.732)	0.703 (0.676–0.731)	0.688 (0.660–0.715)
**Brier score**	0.108 (0.101–0.114)	0.111 (0.104–0.117)	0.111 (0.104–0.117)	0.119 (0.112–0.125)
**DOR**	38.61 (31.16–48.74)	35.20 (28.38–44.22)	34.73 (28.14–43.69)	30.58 (24.88–38.22)
**J**	72.19% (69.46–74.86)	71.15% (68.37–73.82)	70.98% (68.25–73.69)	69.37% (66.57–72.12)
**LR+**	6.68 (5.90–7.69)	5.98 (5.32–6.81)	5.82 (5.19–6.62)	5.55 (4.96–6.30)
**LR-**	0.173 (0.149–0.198)	0.170 (0.146–0.195)	0.168 (0.144–0.193)	0.182 (0.157–0.207)
**PPV**	82.16% (79.93–84.38)	80.47% (78.18–82.74)	80.03% (77.73–82.30)	79.27% (76.92–81.59)
**NPV**	89.34% (87.77–90.84)	89.52% (87.93–91.02)	89.65% (88.09–91.16)	88.88% (87.25–90.45)

Data in parentheses are 95% CIs through bootstrapping. Kappa = Cohen's kappa coefficient. MCC = Matthew's correlation coefficient. DOR = diagnostic odds ratio. J = Youden's J statistic. LR+ = Positive likelihood ratio. LR– = Negative likelihood ratio. PPV = Positive predictive value. NPV = Negative predictive value.

Supplementary Table 11 | Impact of domain shift on AI model performance

	In-domain	Domain-shifted	△(in-domain - domain-shifted)	p-value
**F1 score**	74.54% (69.71–78.97)	70.34% (65.46–74.85)	4.20 (0.85–7.65)	0.0132
**Sensitivity**	90.45% (85.89–94.55)	93.26% (89.33–96.67)	-2.81 (-6.41–0.61)	0.1626
**Specificity**	80.58% (77.04–84.08)	73.28% (69.26–77.18)	7.31 (3.70–11.07)	$<$ 0.0001
**Accuracy**	83.26% (80.37–86.00)	78.69% (75.49–81.74)	4.57 (1.83–7.46)	0.0020
**AUC**	0.944 (0.923–0.963)	0.932 (0.909–0.953)	0.012 (-0.004–0.030)	0.1450
**Kappa**	0.626 (0.564–0.687)	0.552 (0.491–0.613)	0.074 (0.022–0.127)	0.0068
**MCC**	0.648 (0.591–0.704)	0.595 (0.540–0.649)	0.054 (0.005–0.103)	0.0314
**Brier score**	0.115 (0.102–0.128)	0.153 (0.141–0.166)	-0.038 (-0.047–(-)0.029)	$<$ 0.0001
**DOR**	39.31 (24.23–75.24)	37.93 (22.12–81.69)	1.37 (-30.29–26.44)	0.8938
**J**	71.03% (65.43–76.54)	66.54% (61.03–71.85)	4.50 (-0.57–9.70)	0.0808
**LR+**	4.66 (3.91–5.71)	3.49 (3.02–4.10)	1.17 (0.50–2.04)	0.0006
**LR-**	0.119 (0.067–0.175)	0.092 (0.045–0.146)	0.027 (-0.020–0.073)	0.2690
**PPV**	63.39% (57.44–69.26)	56.46% (50.81–62.06)	6.92 (3.07–11.01)	0.0006
**NPV**	95.78% (93.72–97.63)	96.69% (94.77–98.37)	-0.91 (-2.52–0.69)	0.2692

Performance of an AI model on data from a center included during training (in-domain), vs. the performance of an AI model trained on data from other centers (domain-shifted). Both models were evaluated on the same set of 657 cases from the Stockholm center. One of the models was trained on images from 500 cases from the Stockholm center (in-domain), while the other model was trained on images from 500 cases sampled from the other 19 centers (domain-shifted). Data are % (95% CI) or percentage points (95% CI) through bootstrapping, and p-values are based on two-sided non-parametric confidence interval tests. Kappa = Cohen's kappa coefficient. MCC = Matthew's correlation coefficient. DOR = diagnostic odds ratio. J = Youden's J statistic. LR+ = Positive likelihood ratio. LR– = Negative likelihood ratio. PPV = Positive predictive value. NPV = Negative predictive value.

Supplementary Table 12 | Performance of CNN-based model

	Transformer (DeiT)	CNN (ConvNeXt)
**F1 score**	83.50% (81.76–85.14)	82.83% (81.11–84.53)
**Sensitivity**	84.88% (82.73–86.96)	85.16% (82.97–87.24)
**Specificity**	87.30% (85.66–88.94)	85.90% (84.19–87.61)
**Accuracy**	86.32% (85.00–87.59)	85.60% (84.29–86.92)
**AUC**	0.929 (0.919–0.939)	0.925 (0.914–0.935)
**Kappa**	0.718 (0.691–0.745)	0.704 (0.677–0.732)
**MCC**	0.718 (0.691–0.745)	0.705 (0.678–0.733)
**Brier score**	0.108 (0.101–0.114)	0.118 (0.112–0.124)
**DOR**	38.61 (31.16–48.74)	34.98 (28.37–44.12)
**J**	72.19% (69.46–74.86)	71.07% (68.33–73.79)
**LR+**	6.68 (5.90–7.69)	6.04 (5.37–6.89)
**LR-**	0.173 (0.149–0.198)	0.173 (0.149–0.198)
**PPV**	82.16% (79.93–84.38)	80.63% (78.34–82.91)
**NPV**	89.34% (87.77–90.84)	89.37% (87.78–90.88)

Data in parentheses are 95% CIs through bootstrapping. Kappa = Cohen's kappa coefficient. MCC = Matthew's correlation coefficient. DOR = diagnostic odds ratio. J = Youden's J statistic. LR+ = Positive likelihood ratio. LR– = Negative likelihood ratio. PPV = Positive predictive value. NPV = Negative predictive value.

Supplementary Table 13 | Distribution of manufacturers and ultrasound systems

Ultrasound system	Cases
**GE**	3,353 (91.8%)
Voluson E8	1,607 (44.0%)
Voluson E10	1,006 (27.5%)
Voluson E6	325 (8.9%)
Voluson 730	171 (4.7%)
Voluson S10	132 (3.6%)
Voluson I	89 (2.4%)
Voluson S8	18 (0.5%)
Voluson P8	4 (0.1%)
Voluson S6	1 (0.0%)
**Samsung**	175 (4.8%)
WS80A	159 (4.4%)
HS70A	8 (0.2%)
Hera W10	7 (0.2%)
Accuvix A30	1 (0.0%)
**Other**	124 (3.4%)
Philips EPIQ	51 (1.4%)
Mindray DC-70	44 (1.2%)
Toshiba Aplio XG	15 (0.4%)
Esaote MyLab	10 (0.3%)
Mindray M9	1 (0.0%)
Aloka Prosound SSD-5000	1 (0.0%)
Canon Aplio i800	1 (0.0%)
Hitachi (unknown model)	1 (0.0%)

Counts are given with their percentage rate.

Supplementary Table 14 | Breakdown of histological diagnoses at different levels of granularity

2 classes	n	10 classes	n	18 classes	n	Histological	n
Benign	2,224 (60.9%)	Endometrioma	336 (9.2%)	Endometrioma	336 (9.2%)	Endometrioma	336 (9.2%)
		Dermoid	431 (11.8%)	Dermoid	431 (11.8%)	Dermoid	431 (11.8%)
		Other common benign	298 (8.2%)	Simple, Inclusion cyst	106 (2.9%)	Simple cyst	102 (2.8%)
						Inclusion cyst	4 (0.1%)
				Paraovarian cyst	47 (1.3%)	Paraovarian cyst	47 (1.3%)
				Functional cyst	54 (1.5%)	Functional cyst	54 (1.5%)
				Hydrosalpinx, Pyosalpinx, Tubo-ovarian abscess, Peritoneal cyst	91 (2.5%)	Hydrosalpinx	43 (1.2%)
						Pyosalpinx, Tubo-ovarian abscess	32 (0.9%)
						Peritoneal cyst	16 (0.4%)
		Solid benign	153 (4.2%)	Solid benign	153 (4.2%)	Fibroma	114 (3.1%)
						Thecoma	26 (0.7%)
						Myoma	13 (0.4%)
		Cystadeno(fibro)ma	707 (19.4%)	Cystadenoma (serous)	251 (6.9%)	Cystadenoma (serous)	251 (6.9%)
				Cystadenoma (mucinous)	283 (7.7%)	Cystadenoma (mucinous)	283 (7.7%)
				Cystadenofibroma	173 (4.7%)	Cystadenofibroma (serous)	151 (4.1%)
						Cystadenofibroma (mucinous)	22 (0.6%)
		*Rare benign**	66 (1.8%)	*Rare benign**	66 (1.8%)	Struma ovarii	23 (0.6%)
						Brenner tumor	15 (0.4%)
						Endometrioma (decidualized)	13 (0.4%)
						Schwannoma	4 (0.1%)
						Leydig cell tumor	3 (0.1%)
						Other rare benign	8 (0.2%)
		*Ultrasound follow-up**	233 (6.4%)	*Ultrasound follow-up**	233 (6.4%)	*Ultrasound follow-up**	233 (6.4%)
Malignant	1,428 (39.1%)	Borderline (serous)	207 (5.7%)	Borderline (serous)	207 (5.7%)	Borderline (serous)	207 (5.7%)
		Borderline (mucinous intestinal)	100 (2.7%)	Borderline (mucinous intestinal)	100 (2.7%)	Borderline (mucinous intestinal)	100 (2.7%)
		Ovarian cancer (epithelial)	804 (22.0%)	Ovarian cancer (serous), Carcinosarcoma, Tubal cancer	556 (15.2%)	Ovarian cancer (serous)	487 (13.3%)
						Tubal cancer	47 (1.3%)
						Carcinosarcoma	15 (0.4%)
						Other malignancy (epithelial)	7 (0.2%)
				Ovarian cancer (mucinous)	53 (1.5%)	Ovarian cancer (mucinous)	53 (1.5%)
				Ovarian cancer (endometrioid, clear-cell)	195 (5.3%)	Ovarian cancer (endometrioid)	132 (3.6%)
						Ovarian cancer (clear-cell)	63 (1.7%)
		Ovarian cancer (non-epithelial)	116 (3.2%)	Ovarian cancer (non-epithelial)	116 (3.2%)	Granulosa cell tumor	49 (1.3%)
						Yolc sac tumor	11 (0.3%)
						Dysgerminoma	10 (0.3%)
						Sertoli-Leydig cell tumor	10 (0.3%)
						Mixed Germcell tumor	8 (0.2%)
						Other malignancy (non-epithelial)	28 (0.8%)
		Metastasis	201 (5.5%)	Metastasis (colorectal, pancreas)	74 (2.0%)	Metastasis (colorectal)	66 (1.8%)
						Metastasis (pancreas)	8 (0.2%)
				Metastasis (other)	127 (3.5%)	Metastasis (gastric)	41 (1.1%)
						Metastasis (breast)	24 (0.7%)
						Metastasis (endometrial)	7 (0.2%)
						Metastasis (lymphoma)	4 (0.1%)
						Metastasis (other)	51 (1.4%)

Counts are given with their percentage rate. Histological diagnoses were grouped into ten and 18 categories based on histological diagnosis from surgery and sonographic characteristics. *For training, rare benign and ultrasound follow-up cases were, where possible, assigned to one of the other benign histological classes, based on the sonographic characteristics (as assessed by one expert examiner [E.E.]).

Supplementary Table 15 | Center-wise summary of test dataset characteristics, separately for benign and malignant cases

Center	Images per case		Year of examination		Age	
	Benign	Malignant	Benign	Malignant	Benign	Malignant
**Ancona, Italy**	3 (3–4)	4 (3–5)	2020 (2020–2020)	2019 (2019–2020)	49 (38–60)	55 (44–70)
**Athens, Greece**	5 (3–6)	7 (4–10)	2019 (2018–2020)	2019 (2019–2020)	40 (32–49)	46 (33–61)
**Barcelona, Spain**	4 (4–5)	4 (4–5)	2015 (2012–2019)	2014 (2012–2019)	42 (32–47)	49 (40–62)
**Bologna, Italy**	4 (3–6)	6 (4–7)	2018 (2017–2019)	2018 (2016–2019)	44 (33–57)	45 (34–56)
**Brescia, Italy**	4 (3–6)	5 (3–8)	2017 (2016–2019)	2018 (2016–2020)	45 (34–54)	61 (52–67)
**Cagliari, Italy**	4 (3–5)	4 (3–6)	2010 (2010–2011)	2010 (2010–2011)	42 (34–48)	53 (32–62)
**Katowice, Poland**	3 (2–3)	4 (3–6)	2017 (2015–2019)	2017 (2014–2018)	38 (32–42)	51 (45–62)
**Kaunas, Lithuania**	6 (4–8)	6 (4–10)	2020 (2018–2020)	2019 (2018–2020)	41 (31–59)	54 (43–68)
**Lublin, Poland**	4 (3–5)	4 (3–6)	2017 (2016–2020)	2016 (2016–2020)	42 (32–54)	55 (42–64)
**Lund, Sweden**	2 (2–3)	3 (2–4)	2020 (2019–2020)	2020 (2019–2020)	47 (34–64)	62 (47–73)
**Manila, Philippines**	5 (4–7)	4 (3–5)	2019 (2019–2020)	2019 (2019–2019)	32 (24–44)	51 (38–57)
**Milan, Italy**	4 (3–4)	4 (3–6)	2017 (2016–2018)	2017 (2016–2018)	50 (36–55)	52 (45–62)
**Milan 2, Italy**	4 (3–4)	3 (2–4)	2016 (2013–2019)	2016 (2012–2018)	44 (35–59)	55 (47–65)
**Monza, Italy**	3 (3–4)	5 (4–6)	2018 (2018–2019)	2018 (2017–2019)	44 (35–54)	58 (48–69)
**Pamplona, Spain**	3 (2–3)	3 (2–3)	2010 (2009–2011)	2010 (2009–2011)	39 (27–50)	52 (41–67)
**Prague, Czech Republic**	6 (4–6)	4 (4–6)	2020 (2020–2020)	2020 (2019–2020)	56 (39–68)	58 (46–68)
**Prague 2, Czech Republic**	5 (3–5)	5 (4–7)	2014 (2012–2015)	2014 (2012–2014)	36 (31–48)	51 (41–62)
**Stockholm, Sweden**	5 (3–8)	7 (4–9)	2019 (2018–2020)	2019 (2018–2020)	49 (35–59)	58 (44–72)
**Trieste, Italy**	5 (4–7)	8 (6–10)	2020 (2020–2020)	2020 (2019–2020)	52 (40–59)	56 (51–60)
**OVERALL**	**4 (3–5)**	**4 (3–6)**	**2018 (2016–2020)**	**2018 (2016–2020)**	**43 (33–55)**	**55 (44–65)**

Data are median (IQR).

References

[1] Van Calster, B. et al. A calibration hierarchy for risk models was defined: from utopia to empirical data. *J. Clin. Epidemiol.* **74**, 167–176 (2016).

[2] Redmon, J., Divvala, S., Girshick, R. & Farhadi, A. You only look once: unified, real-time object detection. *Proceedings of the IEEE Conference on Computer Vision and Pattern Recognition*, 779–788 (2016).
