# Supplementary material for: International multicenter validation of AI-driven ultrasound detection of ovarian cancer
Source: Nat Med. 2025 Jan 2;31(1):189–96. doi: 10.1038/s41591-024-03329-4 (PMC11750711; doi:10.1038/s41591-024-03329-4)
Supplement: Supplementary file 2 — Reporting Summary [file 41591_2024_3329_MOESM2_ESM.pdf]

nature portfolio

Corresponding author(s): Elisabeth Epstein

Last updated by author(s): Sep 20, 2024

Reporting Summary

Nature Portfolio wishes to improve the reproducibility of the work that we publish. This form provides structure for consistency and transparency in reporting. For further information on Nature Portfolio policies, see our Editorial Policies and the Editorial Policy Checklist.

Statistics

For all statistical analyses, confirm that the following items are present in the figure legend, table legend, main text, or Methods section.

n/a	Confirmed	
☐	☒	The exact sample size (*n*) for each experimental group/condition, given as a discrete number and unit of measurement
☐	☒	A statement on whether measurements were taken from distinct samples or whether the same sample was measured repeatedly
☐	☒	The statistical test(s) used AND whether they are one- or two-sided<br>*Only common tests should be described solely by name; describe more complex techniques in the Methods section.*
☐	☒	A description of all covariates tested
☒	☐	A description of any assumptions or corrections, such as tests of normality and adjustment for multiple comparisons
☐	☒	A full description of the statistical parameters including central tendency (e.g. means) or other basic estimates (e.g. regression coefficient) AND variation (e.g. standard deviation) or associated estimates of uncertainty (e.g. confidence intervals)
☐	☒	For null hypothesis testing, the test statistic (e.g. *F*, *t*, *r*) with confidence intervals, effect sizes, degrees of freedom and *P* value noted<br>*Give P values as exact values whenever suitable.*
☒	☐	For Bayesian analysis, information on the choice of priors and Markov chain Monte Carlo settings
☒	☐	For hierarchical and complex designs, identification of the appropriate level for tests and full reporting of outcomes
☐	☒	Estimates of effect sizes (e.g. Cohen's *d*, Pearson's *r*), indicating how they were calculated

Our web collection on statistics for biologists contains articles on many of the points above.

Software and code

Policy information about availability of computer code

Data collection

Data preparation was conducted using Python (v. 3.8.13) [pandas, Pillow]. SuperAnnotate (https://www.superannotate.com) was used for manual rectangular region of interest selection.

pandas (v. 1.4.3): https://github.com/pandas-dev/pandas
Pillow (v. 8.4.0): https://github.com/python-pillow/Pillow

Data analysis

JASP (v. 0.18.3) and SAS (v. 9.04) [proc mixed] were used for statistical analysis. R (v. 4.3.3) [CalibrationCurves] was used to create the calibration curves. Python (v. 3.8.13) [PyTorch, torchvision, pandas, NumPy, scikit-learn, SciPy, Pillow, Matplotlib, seaborn, timm, YOLO v8] was used for model development, triage simulation, plotting, and model evaluation.

CalibrationCurves: https://github.com/BavoDC/CalibrationCurves

PyTorch (v. 1.11.0): https://github.com/pytorch/pytorch
torchvision (v. 0.12.0): https://github.com/pytorch/vision
pandas (v. 1.4.3): https://github.com/pandas-dev/pandas
NumPy (v. 1.21.6): https://github.com/numpy/numpy
scikit-learn (v. 1.1.1): https://github.com/scikit-learn/scikit-learn
SciPy (v. 1.8.1): https://github.com/scipy/scipy
Pillow (v. 8.4.0): https://github.com/python-pillow/Pillow
Matplotlib (v. 3.5.2): https://github.com/matplotlib/matplotlib
seaborn (v. 0.11.2): https://github.com/mwaskom/seaborn

timm (v. 0.6.7): https://github.com/huggingface/pytorch-image-models
YOLO v8 (v. 8.0.200): https://github.com/ultralytics/ultralytics

For manuscripts utilizing custom algorithms or software that are central to the research but not yet described in published literature, software must be made available to editors and reviewers. We strongly encourage code deposition in a community repository (e.g. GitHub). See the Nature Portfolio guidelines for submitting code & software for further information.

Data

Policy information about availability of data

All manuscripts must include a data availability statement. This statement should provide the following information, where applicable:
- Accession codes, unique identifiers, or web links for publicly available datasets
- A description of any restrictions on data availability
- For clinical datasets or third party data, please ensure that the statement adheres to our policy

Since the examiners did not review cases from their own centres, their assessments will not be made publicly available or shared, as this would expose the identities of the individual examiners. The image data used in this study are not publicly available due to privacy concerns and study-specific data sharing agreements with multiple medical institutions across several countries that prohibit further sharing.
However, researchers interested in conducting analyses or external model validation can submit their code as a dockerized container. We will run this code on our secure servers and provide the results back to the researchers without sharing any raw data. To initiate a request, please contact the corresponding author (E.E.) at elisabeth.epstein@ki.se with a complete study protocol, including a clear research purpose and a detailed description of the proposed analysis. Detailed instructions will be provided upon approval of the request.
Requests from academic investigators without relevant conflicts of interest and intended for non-commercial use will be evaluated within two months based on institutional policies, scientific merit, and the availability of resources required to process the request.
All other data supporting the findings of this study are available within the article and its supplementary information files.

Research involving human participants, their data, or biological material

Policy information about studies with human participants or human data. See also policy information about sex, gender (identity/presentation), and sexual orientation and race, ethnicity and racism.

Reporting on sex and gender	All human participants were female.
Reporting on race, ethnicity, or other socially relevant groupings	No data on race, ethnicity, or other socially relevant groupings were used as no such variables were available.
Population characteristics	Table 1, Extended Data Table 3, Supplementary Fig. 3, Supplementary Table 13, Supplementary Table 14, and Supplementary Table 15 of the paper detailed the population characteristics of the study participants, including information on age, histological diagnosis from surgery, hospital, ultrasound system, and year of ultrasound examination.
Recruitment	No patient recruitment was performed as it was a retrospective study. Participating centres were requested to provide images of at least 50 consecutive malignant cases and at least 50 benign cases, examined just prior to or after each malignant case, to ensure a similar temporal distribution between classes and avoid bias from potential variations in diagnostic practices or equipment over time. This enrichment strategy was designed to ensure an adequate representation of malignant cases, thereby more effectively capturing rare pathologies while minimizing potential biases.
Ethics oversight	The study was approved by the Swedish Ethics Review Authority (Dnr 2020-06919), and by the local ethics review board for each participating centre (outside of Sweden) where required: the Bioethical Committee of the Medical University in Lublin (KE-0254/155/2016, KE-0254/214/2019), the Kaunas Regional Biomedical Research Ethics Committee (BE-2-83), the Brescia Ethics Committee (NP 4591), the Institutional Review Board of the IRCCS Burlo Garofolo (1480/2020), the Ethics Committee of ATS Sardegna (324/2021/CE), the Ethics Committee of the European Institute of Oncology (UID 2505), the Ethics Committee of the Medical University of Silesia, the Ethics Committee of the General University Hospital in Prague (169/22 S-IV), the Ethics Committee of the National and Kapodistrian University of Athens, the Ethics Committee of the Institute for the Care of Mother and Child, the Institutional Review Board of the Rizal Medical Center, the Ethics Committee of the ProVita Medical Centre, Dexeus University Hospital, Clínica Universidad de Navarra.

Note that full information on the approval of the study protocol must also be provided in the manuscript.

Field-specific reporting

Please select the one below that is the best fit for your research. If you are not sure, read the appropriate sections before making your selection.

☒ Life sciences ☐ Behavioural & social sciences ☐ Ecological, evolutionary & environmental sciences

For a reference copy of the document with all sections, see nature.com/documents/nr-reporting-summary-flat.pdf

Life sciences study design

All studies must disclose on these points even when the disclosure is negative.

Sample size	Our initial power analysis, which was based on our plan to compare the AI models with the initial assessments of the ultrasound examiners who generated the images, resulted in a required sample size of 1,600 cases. To account for potential dropout, we initially requested a minimum of 100 cases from each of the 20 participating centres. Our inclusion process exceeded expectations, resulting in a total of 3,652 cases from 19 centres. However, as the examiners' initial assessments had not been systematically documented for most centres, we adjusted our evaluation strategy as detailed in the section titled 'Human examiner review'.<br><br>Participating centres were requested to provide at least 50 benign and 50 malignant cases, in order to allow for a meaningful subgroup analysis at centre-level.
Data exclusions	Exclusion criteria for ultrasound images were: inadequate image quality (e.g., lesions that could not be identified, lesions with blurred margins, and lesions that were only partially visible). Based on this, 4.8% (n = 183/3,840) of the cases (91 benign, 92 malignant) were excluded from the study. The Olbia centre was excluded from testing due to its limited sample size (n = 57) and its small number of malignant cases (n = 8). Fifty-eight (58) cases (Cagliari n = 23, Pamplona n = 12, Trieste n = 23) were excluded from testing as they had not been included in compliance with our criterion on the temporal distribution of examination dates. Due to the lack of histological diagnosis from surgery (conservative management with ultrasound follow-up), 233 cases from the Stockholm centre were excluded from the main analysis and testing, and were instead analysed and reported separately. An additional 644 cases from the Stockholm centre were excluded from testing in order to have a test set of comparable size (n = 300) to those of the other centres and to utilize our reviewer resources efficiently.
Replication	We replicated our experiments 19 times, each time training a model using the same procedure on the majority of the data, and testing on one center. We saw a strong performance for all centres, always outperforming non-expert examiners, and outperforming expert examiners on 17 centres, and on par with expert examiners on one centre.
Randomization	No randomization was performed for test set allocation. We applied a leave-one-centre-out cross-validation scheme, where iteratively each centre in turn was isolated as the test set and the model was given the cases from the remaining centres for training and validation. The allocation of patients to training or validation sets was done at random. For more details, please refer to the Methods section of the manuscript.
Blinding	Investigators were blinded to the test sets until final model selection. No other subjective evaluation which required blinding was performed.

Reporting for specific materials, systems and methods

We require information from authors about some types of materials, experimental systems and methods used in many studies. Here, indicate whether each material, system or method listed is relevant to your study. If you are not sure if a list item applies to your research, read the appropriate section before selecting a response.

Materials & experimental systems

n/a	Involved in the study
☒	☐ Antibodies
☒	☐ Eukaryotic cell lines
☒	☐ Palaeontology and archaeology
☒	☐ Animals and other organisms
☒	☐ Clinical data
☒	☐ Dual use research of concern
☒	☐ Plants

Methods

n/a	Involved in the study
☒	☐ ChIP-seq
☒	☐ Flow cytometry
☒	☐ MRI-based neuroimaging

Plants

Seed stocks	*Report on the source of all seed stocks or other plant material used. If applicable, state the seed stock centre and catalogue number. If plant specimens were collected from the field, describe the collection location, date and sampling procedures.*
Novel plant genotypes	*Describe the methods by which all novel plant genotypes were produced. This includes those generated by transgenic approaches, gene editing, chemical/radiation-based mutagenesis and hybridization. For transgenic lines, describe the transformation method, the number of independent lines analyzed and the generation upon which experiments were performed. For gene-edited lines, describe the editor used, the endogenous sequence targeted for editing, the targeting guide RNA sequence (if applicable) and how the editor was applied.*
Authentication	*Describe any authentication procedures for each seed stock used or novel genotype generated. Describe any experiments used to assess the effect of a mutation and, where applicable, how potential secondary effects (e.g. second site T-DNA insertions, mosiacism, off-target gene editing) were examined.*
